# Integrating molecular QTL data into genome-wide genetic association analysis: Probabilistic assessment of enrichment and colocalization

**DOI:** 10.1371/journal.pgen.1006646

**Published:** 2017-03-09

**Authors:** Xiaoquan Wen, Roger Pique-Regi, Francesca Luca

**Affiliations:** 1 Department of Biostatistics, University of Michigan, Ann Arbor, Michigan, United States of America; 2 Center for Molecular Medicine and Genetics, Wayne State University, Detroit, Michigan, United States of America; 3 Department of Obstetrics and Gynecology, Wayne State University, Detroit, Michigan, United States of America; Vanderbilt University, UNITED STATES

## Abstract

We propose a novel statistical framework for integrating the result from molecular quantitative trait loci (QTL) mapping into genome-wide genetic association analysis of complex traits, with the primary objectives of quantitatively assessing the enrichment of the molecular QTLs in complex trait-associated genetic variants and the colocalizations of the two types of association signals. We introduce a natural Bayesian hierarchical model that treats the latent association status of molecular QTLs as SNP-level annotations for candidate SNPs of complex traits. We detail a computational procedure to seamlessly perform enrichment, fine-mapping and colocalization analyses, which is a distinct feature compared to the existing colocalization analysis procedures in the literature. The proposed approach is computationally efficient and requires only summary-level statistics. We evaluate and demonstrate the proposed computational approach through extensive simulation studies and analyses of blood lipid data and the whole blood eQTL data from the GTEx project. In addition, a useful utility from our proposed method enables the computation of expected colocalization signals using simple characteristics of the association data. Using this utility, we further illustrate the importance of enrichment analysis on the ability to discover colocalized signals and the potential limitations of currently available molecular QTL data. The software pipeline that implements the proposed computation procedures, *enloc*, is freely available at https://github.com/xqwen/integrative.

## Author summary

Genome-wide association studies (GWAS) have been tremendously successful in identifying genetic variants that impact complex diseases. However, the roles of such studies in disease etiology remain poorly understood, primarily because a large proportion of the GWAS findings are located in the non-coding region of the genome. Recent advancements in high-throughput sequencing technology enable the systematic investigation of molecular quantitative trait loci (QTLs), which are genetic variants that directly affect molecular phenotypes (e.g., gene expression, transcription factor binding and DNA methylation). Linking molecular QTLs to GWAS findings intuitively represents an important step for interpreting the biological and clinical relevance of the GWAS results. In this paper, we describe a rigorous and efficient computational approach that assesses the enrichment and overlap between the GWAS findings and molecular QTLs. Importantly, we illustrate that the accurate quantification of overlapping between molecular QTL and GWAS signals requires reliable enrichment estimation. Our proposed approach fully accounts for the intrinsic uncertainty embedded in the association analyses of GWAS and molecular QTL mapping, and it outperforms the existing state-of-the-art approaches. Applying the proposed approach to the GWAS data of blood lipid traits and the whole blood expression QTLs (eQTLs) yields some novel biological insights and also illustrates the potential limitations of the currently available molecular QTL data.

## Introduction

Genome-wide association studies (GWAS) have successfully identified many genomic loci that impact complex diseases and complex traits. Nevertheless, the molecular pathways that connect genetic variants to complex traits are still poorly understood, primarily because a considerable proportion of trait-associated signals are located in the non-coding region of the genome. With recent advancements in high-throughput sequencing technology, systematic investigations of cellular phenotypes have revealed an abundance of non-coding molecular quantitative trait loci (QTLs) [1–4]. Integrating molecular QTL data into GWAS analyses has shown great potential in unveiling the missing links between trait-associated genetic variants and organismal phenotypes [5–7].

In this paper, we focus on a specific type of integrative analysis that aims to assess the overlapping/colocalization of causal GWAS hits and causal molecular QTLs (also known as quantitative trait nucleotides, or QTNs). Following Giambartolomei *et al* [8], we define a GWAS hit and a molecular QTN as being colocalized if a single genetic variant is causally associated with both the complex and molecular traits of interest. Colocalizing genetic variants that jointly affect both molecular and organismal phenotypes provides an intuitive starting point for exploring the role of genetic variants in disease etiology. Taking expression quantitative trait loci (eQTL) mapping as an example, colocalizing an eQTL signal with a GWAS hit naturally suggests that the target gene of the eQTL may play an important role in the molecular pathway of the complex traits. Additionally, other types of available integrative analysis approaches, e.g., *Sherlock* [9], *PrediXcan* [5] and other similar approaches [10, 11], can also benefit from accurate colocalization analysis, either for improved power (as in the case of *Sherlock*) or better interpretation of the inference results (as in the case of *PrediXcan*).

Considering the most common practical setting in which GWAS and molecular QTL data are obtained from two non-overlapping sets of samples, we propose a natural Bayesian hierarchical model for integrating the two types of association data. Specifically, we regard the (latent) association status of each candidate SNP with respect to the molecular phenotype of interest as an SNP-level annotation, and we attempt to quantify the odds of an annotated SNP being causally associated with the complex trait of interest, which is statistically equivalent to evaluating the enrichment level of annotated SNPs in the causal GWAS hits. Subsequently, the resulting enrichment estimates are utilized in the downstream fine-mapping (of GWAS hits) and colocalization analyses. Within our Bayesian hierarchical model, we show that the problems of enrichment estimation, fine-mapping and colocalization testing can be seamlessly solved in a unified inference framework. In addition, our approach is computationally efficient and requires only summary-level data from both molecular QTL mapping and GWAS.

Our proposed method is most similar to the probabilistic model-based approaches *coloc* [8] and *eCAVIAR* [12], which represent the state-of-the-art in the current literature. The advantages of the model-based colocalization analysis methods over the empirical methodologies (e.g., Nica et al [6]) have been fully demonstrated through both rigorous theoretical arguments [8, 13] and carefully constructed simulation studies [12]. In this paper, we show that both *coloc* and *eCAVIAR* can be viewed as special cases of the proposed approach. In particular, both approaches bypass the enrichment analysis by making subjective assumptions on the enrichment levels of molecular QTLs in GWAS signals. In comparison, our approach shares the advantages of both existing approaches, but it enjoys additional flexibility and improved statistical rigor. Most importantly, our approach provides calibrated statistical quantification on colocalized association signals.

## Method

### Model and notation

Without loss of generality, we consider a GWAS of a quantitative trait and describe its associations with *p* candidate SNPs and *n* unrelated samples using a multiple linear regression model,

$$y=\sum_{i=1}^{p}\beta_{i}g_{i}+e,\;e∼N\left(0,\tau^{-1}I\right),$$ (1)

where we assume that both the phenotype and genotypes are centered (the intercept term is therefore exactly 0) and denote the complete collection of genotypes as ***G*** ≔ [***g***_1_, …, ***g***_*p*_]. We further denote the latent binary association status of each SNP *i* by dichotomizing its genetic effect *β*_*i*_, i.e., *γ*_*i*_ = 1 indicates that SNP *i* is genuinely associated (thus, *β*_*i*_ ≠ 0), and *γ*_*i*_ = 0 otherwise. It can be argued that the aim of the GWAS is to make inference of the binary vector ***γ*** ≔ (*γ*_1_, …, *γ*_*p*_). In addition, we assign the standard spike-and-slab prior for each regression coefficient *β*_*i*_ and a flat gamma prior for the residual error variance parameter *τ*.

Suppose that a single quantitative annotation (categorical or continuous) is available for each candidate genetic variant. We integrate the SNP-level annotation into the association analysis by specifying a natural logistic prior for each candidate SNP *i*, i.e.,

$$\log\left[\;\frac{\mathrm{Pr}(\gamma_{i}=1)}{\mathrm{Pr}(\gamma_{i}=0)}\;\right]=\alpha_{0}+\alpha_{1}d_{i}.$$ (2)

In particular, we denote the complete collection of the SNP annotation data as ***d*** ≔ (*d*_1_, …, *d*_*p*_), and we refer to ***α*** ≔ (*α*_0_, *α*_1_) as the enrichment parameter: for a binary annotation, a positive *α*_1_ value indicates that SNPs with the feature have increased odds of being associated with the trait of interest, i.e., the annotated feature is enriched in the trait-associated genetic variants.

In this paper, we consider a special setting in which the annotation is derived from the association analysis of molecular QTL data, namely, (***Y***_*qtl*_, ***G***_*qtl*_). Intuitively, the true association status of each SNP with the molecular phenotype can be naturally incorporated as annotations in Eq (2) for GWAS analysis. However, due to the intrinsic limitations in the molecular QTL mapping, e.g., imperfect power and complication of LD among SNPs, the precise binary association status of each SNP with respect to the molecular phenotype of interest, ***d***, is practically impossible to obtain. Consequently, there is considerable uncertainty in annotating any causal molecular QTN. To fully characterize the uncertainty of the molecular QTL annotation and carry it over into the proposed integrative analysis, we propose embedding a latent covariate model for ***d*** in the prior model (2). Specifically, we consider ***d*** to be an unobserved random vector whose realization is drawn from the following probability distribution:

$$d∼\mathrm{Pr}(d|{Y\;}_{qtl},G_{qtl}).$$ (3)

In particular, we obtain the desired posterior distribution Pr(***d*** ∣ ***Y***_*qtl*_, ***G***_*qtl*_) from a Bayesian multi-SNP association analysis of molecular QTL data [14]. Henceforth, we refer to the distribution Pr(***d*** ∣ ***Y***_*qtl*_, ***G***_*qtl*_) as the “fuzzy” annotation for molecular QTLs.

Based on the proposed Bayesian hierarchical model, we perform statistical inference to address three related problems. First, we aim to estimate the enrichment parameter ***α*** to quantify the enrichment level of molecular QTNs in the causal GWAS hits. Second, we perform Bayesian fine-mapping analysis of GWAS hits accounting for the molecular QTL annotations, and we summarize the results in form of the posterior probability Pr(***γ*** ∣ ***y***, ***G***, ***Y***_*qtl*_, ***G***_*qtl*_). Third, we attempt to evaluate the colocalization of the molecular QTNs and the causal GWAS hits, i.e., for each SNP *i*, we examine whether *γ*_*i*_ = *d*_*i*_ = 1. Within our proposed modeling framework, the colocalization at the single SNP-level is naturally quantified by the posterior probability Pr(*γ*_*i*_ = 1, *d_i_* = 1 ∣ ***y***, ***G***, ***Y***_*qtl*_, ***G***_*qtl*_).

### Impact of enrichment estimation on colocalization analysis

A distinct feature of our proposed integrative analysis framework is the integration of the enrichment estimation in the colocalization analysis. In this section, we illustrate the critical impact of enrichment estimates on the quantitative results of colocalization analysis.

LD is one of the primary factors that complicate the colocalization analysis. This is mainly because of the increasing difficulty in identifying causal SNPs from the association data as the LD between candidate SNPs becomes stronger. Consider a hypothetical example of two perfectly correlated SNPs and assume that they are in complete linkage equilibrium with the remaining candidate SNPs. Suppose that one of the two SNPs is genuinely associated with the molecular phenotype. A well-powered QTL mapping analysis should identify that one of the SNPs is a causal QTN, but there is no further information to distinguish the two. The exact same situation arises if one of the two SNPs (not necessarily the QTN) is genuinely associated with the complex trait. Because of the complete symmetry, the two candidate SNPs also carry identical SNP-level colocalization probabilities and are not identifiable based only on the association data. Nevertheless, a statistical statement can be made regarding the genomic region harboring these two SNPs, and the quantification of such probability can be notably different depending on the enrichment information. If the molecular QTNs are completely irrelevant to the causal GWAS hits, or statistically speaking, ***γ*** and ***d*** are independent (hence, *α*_1_ = 0 in our prior model), we should conclude that there is a 50% chance that the two types of causal associations are overlapped in one of the two SNPs, i.e., the probability that the genomic region harboring a colocalized signal is 0.50. Conversely, if (almost) all the molecular QTNs are indeed causal GWAS hits (hence, *α*_1_ → ∞ in our prior model), we would conclude that, with near certainty, one of the two SNPs is responsible for both genuine associations, i.e., the probability that the region harboring a colocalized signal is approaching 1.0. We would like to note two points from the above hypothetical example: first, in the presence of LD, a regional colocalization probability (RCP) has better practical interpretation than the SNP-level colocalization probability (SCP); second, the enrichment information characterized by *α*_1_ has a profound impact on quantifying RCPs.

Next, we show that the quantified enrichment estimate can be used to calculate the expected number of colocalized association signals based on the proposed prior model without delving into the detailed analysis of individual loci. We denote the marginal (prior) probabilities *p*_*γ*_ ≔ Pr(*γ*_*i*_ = 1) and *p*_*d*_ ≔ Pr(*d*_*i*_ = 1). Based on Eq (2), it follows that

$$\mathrm{Pr}\left(\gamma_{i}=1,d_{i}=1\right)=\frac{p_{\gamma }}{1+\frac{1-p_{d}}{p_{d}}\;e^{-\alpha_{1}}}.$$ (4)

Note that the quantity

$$\rho ≔\mathrm{Pr}\left(d_{i}=1|\gamma_{i}=1\right)=\frac{1}{1+\frac{1-p_{d}}{p_{d}}\;e^{-\alpha_{1}}}$$ (5)

represents the fraction of causal GWAS hits overlapping causal molecular QTNs.

The interplay of *p*_*d*_, *p*_*γ*_ and *α*_1_ with respect to *ρ* can be intuitively understood in some extreme scenarios. For example, if the vast majority of the genome is annotated as molecular QTNs, i.e., if *p*_*d*_ → 1, then *ρ* → 1 and Pr(*γ*_*i*_ = 1, *d*_*i*_ = 1) → *p*_*γ*_. This is because if every SNP in the genome is likely a molecular QTN, then every causal GWAS SNP is also likely a molecular QTN. More generally, the colocalization probability is affected by the enrichment level of molecular QTNs in the GWAS hits. Specifically, if *α*_1_ → ∞, *ρ* → 1 and Pr(*γ*_*i*_ = 1, *d*_*i*_ = 1) → *p*_*γ*_, i.e., all GWAS hits are expected to be molecular QTNs. Alternatively, if *α*_1_ = 0, it follows that *ρ* = *p*_*d*_ and Pr(*γ*_*i*_ = 1, *d*_*i*_ = 1) = *p*_*γ*_ *p*_*d*_, i.e., the two types of associations are mutually independent. Moreover, if molecular QTLs are depleted in the GWAS hits, i.e., *α*_1_ < 0, *ρ* is expected to be < *p*_*d*_.

Furthermore, the prior expected number of colocalized association signals can be simply computed by

$$E\left[\text{Number}\;\text{of}\;\text{colocalized}\;\text{causal}\;\text{variants}\right]=\frac{M\;p_{\gamma }}{1+\frac{1-p_{d}}{p_{d}}\;e^{-\alpha_{1}}},$$ (6)

where *M* represents the total number of SNPs interrogated.

### Background and overview of inference procedure

The exact computation to fit the proposed hierarchical model is intractable. Although approximate computation is theoretically possible using the Markov Chain Monte Carlo (MCMC) algorithm, it does not scale well to genome-wide GWAS and molecular QTL data. Here, we provide the necessary background on the existing computational work and outline the computational procedures to achieve our three inference goals for the integrative analysis.

Assuming that the annotation ***d*** is observed, our previous work [14] proposes a two-stage empirical Bayes procedure to perform accurate and efficient approximate Bayesian inference in the GWAS setting. Briefly, in the first stage, we obtain the maximum likelihood estimate of the enrichment parameter, $\hat{\alpha }$, using an EM algorithm by treating ***γ*** as missing data. Subsequently, in the second stage, we approximate the desired posterior probability Pr(***γ*** ∣ ***y***, ***G***, ***d***) in GWAS analysis by $\mathrm{Pr}(\gamma |y,G,d,\hat{\alpha })$. In addition, and particularly for analyzing GWAS data, we divide the genome into *K* roughly independent LD blocks using the approach described in [15], i.e., ***γ*** = ***γ***_[1]_ ⊕ ***γ***_[2]_ ⊕ ⋯ ⊕ ***γ***_[*K*]_, and further approximate $\mathrm{Pr}(\gamma |y,G,d,\hat{\alpha })$ by $\prod_{i=1}^{K}\mathrm{Pr}\left(\gamma_{\left[i\right]}|y,G,d,\hat{\alpha }\right)$. Within each LD block *i*, $\mathrm{Pr}(\gamma_{\left[i\right]}|y,G,d,\hat{\alpha })$ is then computed using the deterministic approximation of posteriors (DAP) algorithm. Among the two variants of the DAP algorithm described in [14], the adaptive DAP algorithm implements a fully automated Bayesian multi-SNP analysis procedure. Conversely, the DAP-1 algorithm further assumes at most a single causal association within the LD block of interest, but it achieves even more efficient computation and requires only summary-level statistics from the GWAS data.

With the added latent covariate model (3), the computational challenge becomes even greater. We extend our existing empirical Bayes framework into a three-stage procedure to explicitly account for the fuzzy annotation of ***d***. The first stage focuses on finding the MLE $\hat{\alpha }$ in the presence of missing data ***d***. In the second stage, we approximate Pr(***γ*** ∣ ***y***, ***G***, ***Y***_*qtl*_, ***G***_*qtl*_) by $\mathrm{Pr}(\gamma |y,G,{Y\;}_{qtl},G_{qtl},\hat{\alpha })$ to conduct fine-mapping of GWAS signals incorporating the annotation of molecular QTNs. The particular emphasis in this step is to construct the SNP-level priors accounting for the uncertainties of molecular QTLs. In the last stage, we use the results from the previous stages to approximate the SNP-level posterior probability Pr(*γ_i_* = 1, *d_i_* = 1 ∣ ***y***, ***G***, ***Y***_*qtl*_, ***G***_*qtl*_) by $\mathrm{Pr}(\gamma_{i}=1,d_{i}=1|y,G,{Y\;}_{qtl},G_{qtl},\hat{\alpha })$ and the corresponding RCPs for colocalization analysis. (As a notational footnote, conditional on $\hat{\alpha }$, the SNP-level *γ*_*i*_ and *d*_*i*_ depend only on one relevant molecular phenotype and its corresponding genotypes rather than the full collection of the molecular phenotypes. We keep the current notation for the consistency of the presentation.) The subsequent sections provide the statistical and computational details within each stage.

We implement the computational procedure outlined above in the software package *enloc* (Enrichment estimation aided colocalization analysis), which is freely available at https://github.com/xqwen/integrative. Note that the computational procedure requires only summary-level information from both the molecular QTL data and GWAS data.

### Enrichment analysis of molecular QTLs in GWAS hits

The primary objective of the enrichment analysis is to estimate the hyper-parameter ***α*** given the observed summary statistics from GWAS and the fuzzy annotation of molecular QTLs. Recall that if the binary molecular QTL annotation is indeed known, then the EM algorithm that we previously described [14, 16] can be directly applied to obtain the maximum likelihood estimate of ***α***. With incomplete information on annotation data, we adopt a principled statistical strategy in missing data inference known as *multiple imputation* [17, 18]. Specifically, the multiple imputation procedure creates *m* complete data sets by filling in, i.e., imputing, the missing entries of the binary annotation data. The imputed data sets are then individually analyzed using the existing EM algorithm, and the distinct estimates of $\hat{\alpha }$ from multiple imputed data sets are combined into a final estimate using a set of rather simple rules (section S.1 in S1 Text). The key to implementing this strategy is to impute the annotations, which, in our case, is achieved by sampling from the posterior distribution Pr(***d*** ∣ ***Y***_*qtl*_, ***G***_*qtl*_).

According to the missing data theory, the ideal probability distribution to impute ***d*** is Pr(***d*** ∣ ***Y***_*qtl*_, ***G***_*qtl*_, ***y***, ***G***), i.e., the imputation of ***d*** should also be conditioned on the observed GWAS data. The proposed imputation distribution represents a simplified approximation and essentially assumes the independence between ***d*** and GWAS data, which is because Pr(***d*** ∣ ***Y***_*qtl*_, ***G***_*qtl*_) = Pr(***d*** ∣ ***Y***_*qtl*_, ***G***_*qtl*_, ***y***, ***G***) if and only if *α*_1_ = 0. Consequently, imputing from this simplified distribution (or more generally, imputing without the consideration of GWAS data) leads to conservative point estimates that are shrunk toward 0. (This is because each imputed data set is generated as if *α*_1_ is set to 0 *a priori*.) In practice, the underestimation of the true *α*_1_ under the simplified imputation distribution can be noticeable if the true *α*_1_ is much larger than 0 (which is evident in some of our simulation scenarios). Despite this shortcoming, we choose to work with the simplified imputation distribution, Pr(***d*** ∣ ***Y***_*qtl*_, ***G***_*qtl*_), mainly because of its attractive computational property. For example, it can be obtained by a single run of genetic association analysis based solely on the molecular QTL data and applied in the integrative analysis of any GWAS data. In comparison, Pr(***d*** ∣ ***Y***_*qtl*_, ***G***_*qtl*_, ***y***, ***G***) is specific to each GWAS-molecular QTL data set pair, and its computation is considerably more expensive if not practically impossible. Importantly, the empirical evidence from the simulation studies suggests that the bias of the enrichment estimate due to the use of the simplified imputation distribution has non-significant impacts on the results of downstream fine-mapping and colocalization analyses.

The number of imputed data sets (*m*) necessary for reliable estimation has been systematically studied in the missing data theory. The common consensus in the statistical literature is that *m* should be determined by the percentage of missingness, and various theoretical and empirical studies [19, 20] roughly agree that 20 imputations are required for 10% to 30% missing information and that 40 imputations are required for 50% missing information. Although the true annotation ***d*** is completely unobserved in our context, we are certain that *d*_*i*_ = 0 for the vast majority of the candidate SNPs based on inspection of the posterior distribution Pr(***d*** ∣ ***Y***_*qtl*_, ***G***_*qtl*_). In fact, by examining the analysis results of *cis*-eQTLs from the GTEx whole blood data, we find that there are only ∼ 1.5% *cis* candidate SNPs with a posterior inclusion probability ≥ 0.01. Guided by this empirical evidence, we choose to impute *m* = 25 QTL data sets for each analysis. (We have also experimented with 50 and more imputed data sets in the simulations, and the inference results are virtually unchanged.)

Additionally, we observed that detectable GWAS hits and eQTLs (with currently available sample sizes) are both relatively sparse in practice, which can lead to large variances for the estimated enrichment parameter *α*_1_. To illustrate this point, we consider that both ***γ*** and ***d*** are observed; it is then trivial to estimate ${\hat{\alpha }}_{1}$ using a 2 × 2 contingency table. Because each binary vector contains only very few non-zero entries, the resulting contingency table is extremely imbalanced. Consequently, the variance of ${\hat{\alpha }}_{1}$ (approximately equal to the inverse of the smallest cell count) can be large, and the point estimate can be unstable. To stabilize the estimate of the enrichment parameter, we modify the original EM algorithm and apply an *l*_2_ penalty with a shrinkage parameter *λ* in the M-step to shrink the estimate toward 0. This strategy is informed by the statistical principle of “variance-bias trade-off”. Alternatively, this can be viewed as assigning a N(0, 1/*λ*) prior to *α*_1_. In practice, we select *λ* in a data-driven manner by assessing the degree of imbalance of the unobserved contingency table (section S.2 of S1 Text), which assigns stronger penalties for larger degrees of imbalance.

### Fine-mapping incorporating molecular QTL annotations

Given the point estimate of the enrichment parameter, we adopt an empirical Bayes procedure to infer the true association status, ***γ***, for all SNPs in GWAS. Specifically, we compute $\mathrm{Pr}(\gamma |y,G,{Y\;}_{qtl},G_{qtl},\hat{\alpha })$ as an approximation of the desired quantity Pr(***γ*** ∣ ***y***, ***G***, ***Y***_*qtl*_, ***G***_*qtl*_) [21]. In addition, we apply the same divide-and-conquer strategy described in [14] by decomposing the genome into *K* non-overlapping LD blocks [15] and performing independent Bayesian fine-mapping analysis within each LD block. Finally, we summarize the evidence of association for each SNP by its posterior inclusion probability (PIP), i.e., $\mathrm{Pr}(\gamma_{i}=1|y,G,{Y\;}_{qtl},G_{qtl},\hat{\alpha })$.

To account for the uncertainty of the association status of molecular eQTLs, we construct a two-component mixture prior for each SNP, i.e.,

$$\mathrm{Pr}\left(\gamma_{i}=1|{Y\;}_{qtl},G_{qtl},\hat{\alpha }\right)=\frac{e^{{\hat{\alpha }}_{0}}}{1+e^{{\hat{\alpha }}_{0}}}\cdot \left(1-\delta_{i}\right)+\frac{e^{{\hat{\alpha }}_{0}+{\hat{\alpha }}_{1}}}{1+e^{{\hat{\alpha }}_{0}+{\hat{\alpha }}_{1}}}\cdot \delta_{i},$$ (7)

where *δ_i_* ≔ Pr(*d_i_* = 1 ∣ ***Y***_*qtl*_, ***G***_*qtl*_) denotes the PIP of SNP *i* being a causal molecular QTN.

Because the vast majority of the LD blocks harbor no noteworthy association signals for any given complex trait, we follow the common practice in the GWAS analysis and adopt a pre-screening procedure to identify LD regions that are potentially interesting for fine-mapping analysis. Specifically, we use a rigorous Bayesian false discovery rate (FDR) control procedure [22] to screen and select LD blocks for the subsequent fine-mapping analysis. This procedure is typically less conservative (and hence more powerful) than the commonly applied empirical procedures based on the combination of single-SNP testing and the Bonferroni correction. For each identified LD block, we then proceed to perform fine-mapping analysis using the DAP algorithm.

We find that the DAP-1 algorithm is practically adequate for fine-mapping most LD blocks in GWAS data, as we observe that the vast majority of the selected LD blocks harbor no more than a single association signal. Even if multiple GWAS signals co-exist in a single LD block, the DAP-1 algorithm can still be applied when aided by the conditional analysis approach proposed by [23]. Alternatively, the adaptive DAP algorithm, which enables fully automated multi-SNP analysis, can be conveniently applied in this context, even with summary-level statistics (section S.5 of S1 Text). However, there is an increased computational cost. Our simulation study shows that the adaptive DAP algorithm slightly outperforms the DAP-1 algorithm, which confirms the benefit of multi-SNP analysis. Nevertheless, we conclude that the results obtained from the two variants of the DAP algorithm are quite comparable in our simulation studies using realistically generated GWAS data. By default, in this paper, we apply the DAP-1 algorithm for the fine-mapping procedure, and we only re-examine the noticeable loci (e.g., those identified in the subsequent colocalization analysis) using the adaptive DAP algorithm.

### Colocalization analysis of GWAS and molecular QTL data

Given the PIP from the fine-mapping analysis, the SNP-level colocalization probability (SCP) for SNP *i* can be obtained as

$$\begin{array}{cc} & \mathrm{Pr}(\gamma_{i}=1,\delta_{i}=1|y,G,{Y\;}_{qtl},G_{qtl},\hat{\alpha })\\ & =\mathrm{Pr}\left(\gamma_{i}=1|y,G,{Y\;}_{qtl},G_{qtl},\hat{\alpha }\right)/\left[1+\frac{1-\delta_{i}}{\delta_{i}}\cdot \frac{1+e^{{\hat{\alpha }}_{0}+{\hat{\alpha }}_{1}}}{e^{{\hat{\alpha }}_{1}}+e^{{\hat{\alpha }}_{0}+{\hat{\alpha }}_{1}}}\right] \end{array}$$ (8)

by solving a simple linear system (section S.3 of S1 Text).

Based on the discussion in the previous sections and following Gaun and Stephens [24] and Wen *et al* [16], we propose computing a *regional colocalization probability*, or RCP, by summing up the SNP-level colocalization probabilities (SCPs) of correlated SNPs within an LD block that harbors a single GWAS association signal. RCP is naturally interpreted as the probability of a genomic region harboring a colocalized signal. We recommend reporting both RCPs and SCPs in colocalization analysis. In practice, we only compute RCPs for the same LD blocks that are identified by the pre-screening step in the fine-mapping analysis. The rationale is simple: we do not expect an LD block to harbor a colocalized signal if it is unlikely to harbor a GWAS signal.

**Fig 1:**
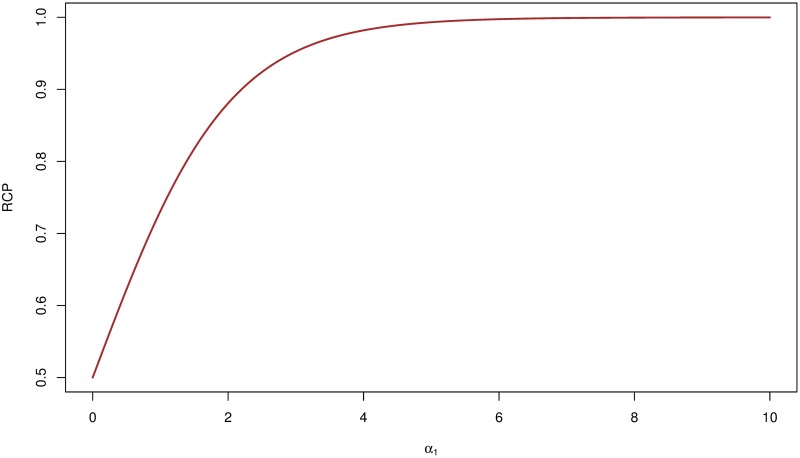
Functional relationship between RCP and enrichment parameter *α*_1_ in a hypothetical example. We consider two perfectly linked SNPs: one is causally associated with the molecular phenotype of interest, and one is causally associated with the complex trait of interest. Assuming that the two SNPs are in complete linkage equilibrium with other SNPs, the plot shows the functional relationship of the RCP value with respect to the enrichment parameter. Note that we should conclude that the two association signals are colocalized (RCP → 1) only if the enrichment level is sufficiently high. It is also theoretically possible that the RCP ≤ 0.5 if the molecular eQTLs are depleted in the GWAS hits, i.e., *α*_1_ < 0.

To demonstrate, we apply Eq (8) in our previously stated hypothetical example of two perfectly linked candidate SNPs. Under the assumption, it follows that at the SNP level, *δ*_1_ = *δ*_2_ = 0.5 and $\mathrm{Pr}\left(\gamma_{1}=1|y,G,{Y\;}_{qtl},G_{qtl},\hat{\alpha }\right)=\mathrm{Pr}\left(\gamma_{2}=1|y,G,{Y\;}_{qtl},G_{qtl},\hat{\alpha }\right)=0.50$. From Eq (8), it is evident that the SCPs of the two SNPs are also identical with the actual value depending on ${\hat{\alpha }}_{1}$: as ${\hat{\alpha }}_{1}\to 0$, both take a value of 0.25 (hence, RCP = 0.50), whereas when ${\hat{\alpha }}_{1}\to \infty$, both take a value of 0.50 (hence, RCP = 1.0). More generally, we show the functional relationship between RCP and the *α*_1_ values in Fig 1, which illustrates the quantitative impact of the enrichment estimation on the probabilistic assessment of colocalized signals.

#### Connection to existing probabilistic colocalization approaches

In this section, we show that Eq (8) represents a generalization of existing probabilistic approaches for colocalization analysis, namely, *eCAVIAR* and *coloc*. In particular, we argue that both of those approaches bypass enrichment estimation by making explicit assumptions on the enrichment parameters.

If the molecular QTNs and causal GWAS hits are assumed to be independent *a priori*, i.e., *α*_1_ is restricted to 0, then the prior for each SNP in GWAS becomes irrelevant to the molecular QTL data, and Eq (8) can be subsequently simplified to

$$\begin{array}{c} \mathrm{Pr}\left(\gamma_{i}=1,\delta_{i}=1|y,G,{Y\;}_{qtl},G_{qtl},\hat{\alpha }\right)=\mathrm{Pr}\left(\gamma_{i}=1|y,G,{\hat{\alpha }}_{0}\right)\cdot \mathrm{Pr}\left(d_{i}=1|{Y\;}_{qtl},G_{qtl}\right), \end{array}$$ (9)

which coincides with the colocalization posterior probability (CLPP) proposed in *eCAVIAR*.

In section S.4 of S1 Text, we provide the derivation of the *coloc* model as a special approximation from our generalized modeling framework given the additional simplifying assumptions. Noticeably, *coloc* assumes that at most a single GWAS hit and/or a single QTN are located in the LD regions of interest. More importantly, it requires the user to specify the priors for *p*_1_ ≔ Pr(*γ*_*i*_ = 1, *d*_*i*_ = 0), *p*_2_ ≔ Pr(*γ*_*i*_ = 0, *d*_*i*_ = 1) and *p*_12_ ≔ Pr(*γ*_*i*_ = 1, *d*_*i*_ = 1). We show that these quantities can be equivalently parametrized within our modeling framework. For example,

$$\alpha_{0}=\log\left[\frac{p_{1}}{1-p_{1}-p_{2}-p_{12}}\right],\;\;\alpha_{1}=\log\left[\frac{p_{12}\;\left(1-p_{1}-p_{2}-p_{12}\right)}{p_{1}\;p_{2}}\right].$$ (10)

Moreover, note that the set of priors required by *coloc* also implicitly induces the marginal frequencies of causal GWAS hits (i.e., Pr(*γ*_*i*_ = 1)) and eQTNs (i.e., Pr(*d*_*i*_ = 1)). Many have reported the sensitivity of the analysis results with respect to the subjective prior specification. We examine the performance of *coloc* when the priors are misspecified using our simulated data (section S.4 of S1 Text). In brief, we find that severe prior misspecifications can lead to inferior performance for ranking potential colocalized signals and inflation of type I errors in the setting of hypothesis testing. In comparison, our proposed approach eliminates the subjective prior quantification and improves the overall robustness in colocalization analysis.

#### Bayesian hypothesis testing of colocalization

In colocalization analysis, it is occasionally of interest to test the following hypothesis:

*H*_0_: Genomic region *i* does not contain a colocalized signal
 *vs*.
*H*_1_: There is a colocalized association signal in region *i*

for each locus *i*. Here, we show that the above hypothesis testing problem can be conveniently solved through the posterior inference within the proposed Bayesian framework.

Given a set of rejected hypotheses *M*, the Bayesian false discovery rate (FDR) can be intuitively estimated by

$$\text{FDR}\left(M\right)=\frac{\sum_{i\in M}\left(1-{\text{RCP}}_{i}\right)}{\left|M\right|},$$

where |*M*| denotes the number of rejected hypotheses [22, 25, 26]. Therefore, at a pre-defined FDR level *α*, the Bayesian FDR control procedure simply ranks all candidate loci according to increasing values of (1 − RCP_*i*_) and rejects the null hypotheses for the largest set *M*, where

$$\frac{\sum_{i\in M}\left(1-{\text{RCP}}_{i}\right)}{\left|M\right|}\leq \alpha .$$

## Results

### Ethics statement

This study uses third party datasets and no additional ethics approval was needed.

### Simulation study

First, we perform simulation studies to benchmark the performances of the proposed enrichment and colocalization analysis approaches.

We design the simulation scheme to generate realistic single SNP association *z*-statistics that are similar to the observed GWAS results. Specifically, we select real genotypes of 2.7 million overlapping SNPs used by both Wood *et al* [27] and the GTEx project from the European samples from the 1000 Genomes Project. For each SNP, we obtain its binary eQTL annotation by drawing from the posterior distribution of GTEx whole blood *cis*-eQTLs the GTEx. This particular posterior distribution is obtained by performing multi-SNP fine-mapping of the GTEx whole blood data via the adaptive DAP algorithm [14]. In total, we roughly annotate ∼ 6,000 SNPs per simulation. We then simulate the association status of each SNP *i* (*γ*_*i*_) by drawing from a Bernoulli distribution whose success rate is determined by the logistic model (2) with pre-determined *α*_0_ and *α*_1_ values. Subsequently, a quantitative trait is simulated using a standard multiple linear regression model for which the residual error variance is set to 1, and the effect size of each causal SNP is drawn from a N(0, *ϕ*^2^) distribution. Finally, we compute the single SNP association *z*-statistic for each SNP as the input for both the enrichment and the colocalization analyses. Although the sample size in the 1000 Genomes Project European panel is limited, we are able to adjust the values of *α*_0_ (which determines the prevalence of the causal associations) and *ϕ* (which determines the signal-to-noise ratio of the genetic effects) to roughly match the *z*-value distributions from the available large-scale GWAS meta-analysis. In particular, we estimate *α*_0_ and *ϕ* by analyzing the height data reported in Wood *et al* [27], and we set *α*_0_ = −8.4 and *ϕ* = 0.4. Consequently, the distributions of the simulated *z*-statistics closely resemble the actual observed GWAS height data (S1 Fig). We vary the value of *α*_1_ across simulations for different levels of enrichment.

#### Evaluation of enrichment analysis

We examine the performance of the proposed inference procedure in estimating the enrichment parameter *α*_1_. In particular, we vary the true *α*_1_ value in the range of 0.0 to 5.0 in the simulations. For each *α*_1_ value, we simulate 100 data sets and estimate *α*_1_ for each simulated data set using the proposed multiple imputation approach.

To benchmark the performance of the proposed approach, we also estimate *α*_1_ using two unrealistic approaches with added information. The first approach represents the best case scenario in which the true association indicators of each SNP in GWAS and eQTL mapping, i.e., *γ*_*i*_ and *d*_*i*_, are assumed to be observed. In this case, *α*_1_ is trivially estimated using a 2 × 2 contingency table. The second approach assumes that the association indicator of GWAS, *γ*_*i*_, is unobserved but that the true eQTL annotation for each SNP, *d*_*i*_, is known, which presents a type of integrative analysis considered in our previous work [14]. In this scenario, we apply the EM-DAP1 algorithm implemented in the software package TORUS [22] to estimate *α*_1_. Note that both of these approaches require additional information that is practically unattainable. Nevertheless, the results from these analyses highlight the intrinsic difficulty of the task and the theoretical ceiling of any realistic computational approach.

We also include two additional *ad hoc* imputation strategies for enrichment estimation for comparison. The first strategy applies “mean imputation”, i.e., for each SNP, we regard the marginal PIP of each SNP (which is also the posterior mean of the corresponding *d*_*i*_ value) as an observed continuous annotation. The second strategy, known as “best SNP imputation”, annotates the best associated *cis* candidate SNP of each eGene (i.e., the gene identified to harbor at least one causal eQTL) as the causal eQTN.

We compute the root-mean-square error (RMSE) for all methods to evaluate the overall accuracy of the corresponding point estimates, which is most relevant for the downstream analysis. In addition, we plot the averaged point estimates and corresponding standard errors from each simulated *α*_1_ value, which helps virtually dissect the relative variance and the bias of the point estimates from each estimation method. The results from various approaches are summarized in Table 1 and S2 Fig.

**Table 1:** Evaluation of the accuracy of various enrichment estimation approaches. Using the simulated data sets, we compute the root-mean-square errors (RMSEs) to measure the precision of the point estimates obtained by different approaches. The methods denoted by * use added information that is unattainable in practice. The methods denoted by ^†^ do not apply shrinkage to the enrichment estimate. The proposed multiple imputation approach yields the best accuracy among approaches that are practically applicable.

Method	RMSE
Best case*^,^^†^	0.374
True annotation*	0.812
Multiple imputation	1.041
True annotation (no shrinkage) *^,^^†^	1.153
Best SNP annotation	1.474
Mean imputation^†^	2.942

Importantly, we note that when the enrichment level is low, the accurate estimation of *α*_1_ is difficult even in the best case scenario: the point estimates show large variance even when the true values of *γ*_*i*_ and *d*_*i*_ are known. In comparison, we observe that the estimates obtained using the proposed approach are significantly stabilized by applying the proposed adaptive shrinkage. As *α*_1_ increases to relatively large values (> 3.0), the effects of shrinkage gradually diminish for all approaches: in the case that the true QTL annotation is known, the estimates become practically unbiased, although for the multiple imputation procedure, the resulting estimates are still notably biased toward 0, largely due to the simplified imputation distribution. Nonetheless, we note that the degree of bias has minimal impact on the subsequent colocalization analysis. The results clearly indicate that the multiple imputation procedure outperforms the two alternative *ad hoc* imputation approaches. The difference in performance between the proposed approach and the best SNP imputation is generally expected because the latter ignores the uncertainty due to LD and the potential multiple independent eQTLs within a gene. We observe that the mean imputation approach consistently (and occasionally severely) overestimates *α*_1_ for large *α*_1_ values, which becomes a serious concern for the downstream colocalization analysis. (We provide some theoretical discussion on the potential contributing factors to this phenomenon in section S.6 of S1 Text). Note that the use of mean imputation in our scenario is different than the case of mean genotype imputation commonly applied in GWAS. This is because in GWAS, there is generally a stringent threshold for filtering out inaccurate imputation for downstream association analysis, and the resulting mean imputations accurately resemble the true genotypes. Conversely, in our case, the PIPs are considerably less accurate representations for the true eQTL association status, particularly for QTNs (e.g., they are rarely close to 1 in general due to the widespread LD).

Furthermore, we examine the statistical performance of the proposed approach for testing the null hypothesis

$$H_{0}:\;\alpha_{1}=0$$

by inspecting the corresponding estimate of the 95% confidence interval from each simulated data set. Our results indicate that the testing results based on the proposed multiple imputation approach properly control type I error at the 5% level with the actual type I error rate = 0.01. Although it achieves nearly perfect power as the true *α*_1_ ≥ 4, it only displays modest power (53%) for *α*_1_ = 3 and little power for smaller *α*_1_ values. Furthermore, despite the point estimates being downward biased, we observe that the proposed multiple imputation procedure provides excellent 95% interval estimates in the range of the *α*_1_ values examined experimentally: the coverage probability reaches 94.8%.

Finally, the benchmarked computational time indicates that the proposed multiple imputation approach is highly efficient. We take advantage of the fact that the multiple imputation scheme is parallelizable and analyze each simulated data set on 8 simultaneous threads. Consequently, each enrichment analysis only takes approximately 4 to 5 minutes of real computing time.

#### Evaluation of colocalization analysis

To evaluate the performance of the colocalization analysis, we focus on the simulation setting of *α*_1_ = 4, which is close to our enrichment estimate of blood eQTLs in HDL GWAS hits from the real data. For each simulated data set, we perform the proposed colocalization analysis using two different fine-mapping strategies. The first strategy utilizes the individual-level genotype data from GWAS and obtains the GWAS PIPs by multi-SNP fine-mapping using the adaptive DAP algorithm. The second strategy assumes at most one causal GWAS hit within each LD block and computes the PIPs using the DAP-1 algorithm based only on the single-SNP association *z*-statistics. To evaluate the impact of the (imperfect) enrichment parameter estimate, we separately use the true and estimated (*α*_0_, *α*_1_) values (by multiple imputations) to construct the SNP-level prior Eq (7) for fine-mapping when applying each strategy. For comparison, we perform the colocalization analysis of the simulated data assuming independence of molecular eQTLs and GWAS hits (i.e., set *α*_1_ = 0 in prior model (2)), which is essentially the enrichment assumption made by *eCAVIAR*. In all cases, we compute the RCPs for all the pre-defined LD blocks in each simulated dataset. Additionally, we run the software package *coloc* on the simulated data. Because its setup is very different from the aforementioned approaches, particularly in its use of eQTL data, without diluting our main messages on the importance of enrichment estimation, we summarize its performance in section S.4 of S1 Text.

**Fig 2:**
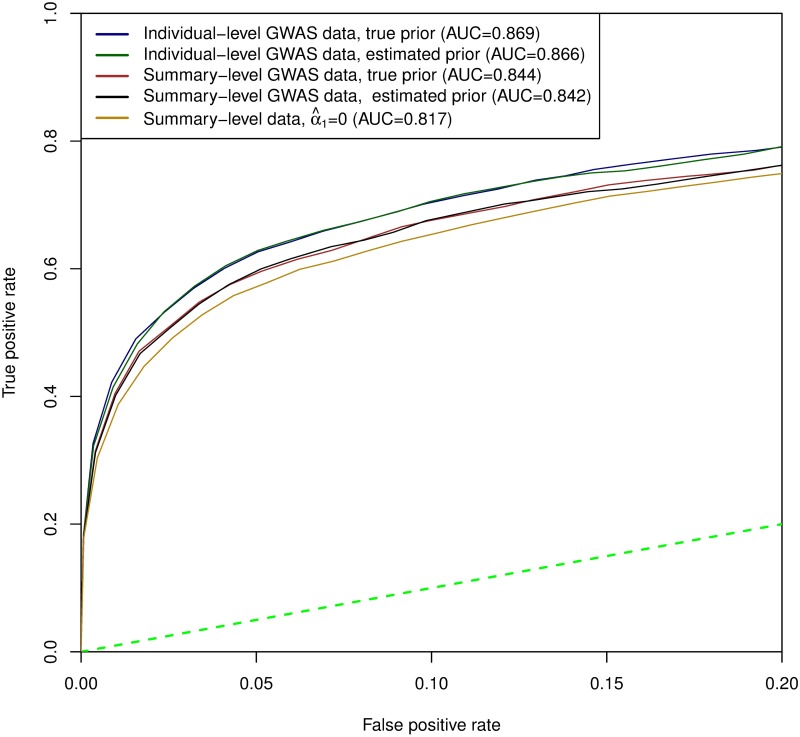
ROC curves for various colocalization analysis schemes in simulation studies. ROC curves evaluate the ranking of the LD blocks that potentially harbor colocalized association signals. The dotted green line represents the 45 degree diagonal line. All schemes perform decently in the simulations. Notably, the inaccuracy of the estimated enrichment parameters from the proposed multiple imputation procedure does not appear to have a significant impact on the overall performance of the colocalization analysis. However, the difference becomes highly visible for the case where *α*_1_ is set to 0. In addition, multi-SNP analysis in GWAS also improves the performance of the colocalization analysis.

First, we construct receiver operating characteristic (ROC) curves to simultaneously evaluate the sensitivity and specificity of various colocalization analysis approaches. Specifically, we classify an LD block as harboring a colocalized signal if the corresponding RCP is greater than a pre-defined threshold. We vary the threshold from 1 to 0 to construct the ROC curve for each analysis scheme. The results are presented in Fig 2, which highlights the performance of each examined approach as the corresponding false positive rates (FPR) ≤0.20. In summary, we find that all approaches yield reasonably decent results in identifying true colocalized signals while controlling for false positives (i.e., they are all well above the 45 degree diagonal line). In particular, we note that i) the ability to identify multiple independent GWAS hits within an LD block (i.e., in the adaptive DAP algorithm) slightly improves the performance of colocalization analysis, but the DAP-1 algorithm performs adequately; ii) the downward bias in the enrichment parameter estimates from the proposed multiple imputation approach has very little impact on the colocalization analysis *at any given FPR threshold*; and iii) neglecting the enrichment analysis only yields slightly worse colocalization results.

**Fig 3:**
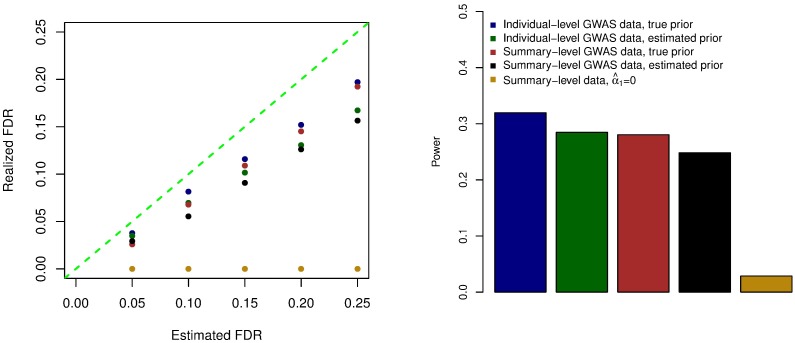
Evaluation of type I error rate and power for various colocalization analysis schemes in simulation studies. This exercise helps to evaluate the calibration of the reported RCPs from various analysis schemes. Better calibrated RCPs result in less conservative control of the type I errors and improved power. Note that the underestimation of ${\hat{\alpha }}_{1}$ results in noticeable, but not substantial, power loss. The results indicate that the RCPs are better calibrated for more accurate enrichment estimates and/or the use of multi-SNP analysis in GWAS.

Note that the ROC curves rely only on the ranking of the corresponding RCPs and are invariant under the rank-preserving transformations. To investigate the calibration of the RCPs reported by various analysis schemes, we further examine the Bayesian FDR control of colocalization analysis based on RCPs. Fig 3 shows the comparison of the estimated FDRs and the realized FDRs for all analysis schemes in the simulations. All approaches (conservatively) control the desired FDR levels; however, the scheme assuming *α*_1_ = 0 is extremely conservative, where the realized FDRs are nearly 0 and the power is significantly lower than all the other competing schemes. We therefore conclude that the accurate enrichment estimation has a critical impact on the quantification of the colocalized signals. In general, we find that the power to detect colocalized association signals is low across different schemes, i.e., < 40% at the 20% FDR level (Fig 3). Because our simulated data closely mimic the reality of the currently available GWAS and eQTL data, we attribute the lack of power reflected by these simulations to the limitations of the currently available genetic association data. (This point will be further demonstrated by the power calculation in the real data applications.)

Our benchmark also indicates that the proposed procedure is highly efficient. The combined computational time for the fine-mapping and colocalization procedure is typically 10 to 20 minutes, depending on the abundance of the GWAS signals.

Taken together, we conclude that the estimation of the enrichment parameters embedded in the prior model (2) impacts both the ranking and calibration of locus-level posterior probabilities for colocalization. According to the ROC curves, the impact on the ranking can be relatively insignificant with respect to non-trivial deviation from the truth for the enrichment parameter. However, the calibration of the colocalization probabilities is considerably more sensitive to such deviation, as evidenced by the power and the realized FDRs in the hypothesis testing of colocalization.

### Integrative analysis of blood eQTL and lipid GWAS data

To demonstrate the proposed computational approach in a practical setting, we perform an integrative analysis of the eQTL data from the GTEx project [1] and the blood lipid data originally reported in Teslovich *et al* [7]. The blood lipid data consist of meta-analysis results of four quantitative traits, namely, low-density lipoprotein (LDL) cholesterol, high-density lipoprotein (HDL) cholesterol, triglycerides (TG) and total cholesterol (TC), with an aggregated sample size of ∼ 100,000. We obtain the version of single-SNP association *z*-statistics for the four traits re-analyzed by Pickrell [28], where additional *z*-statistics for untyped SNPs are imputed according to the 1000 Genomes Project phase I panel. In total, the complete data set contains *z*-scores of ∼ 6.1 million SNPs per trait. For most of our analysis, we focus on the *cis*-eQTL data from the whole blood in the recent release (version 6) of the GTEx project. The selection of the whole blood is informed by the consensus of multiple independent enrichment analysis approaches (GTEx consortium, manuscript in prep.) to determine the relevant tissues for the blood lipid traits. In addition to biological relevance, we suspect that one of the driving factors is that the whole blood is one of the GTEx tissues with the largest sample size (338) in the current release of the data; it therefore has better power to detect *cis*-eQTLs with small to modest effects. The SNPs that are not directly genotyped are also imputed according to the same 1000 Genomes panel by the GTEx consortium. We perform the Bayesian multi-SNP fine-mapping analysis for the GTEx whole blood data using the adaptive DAP algorithm and generate the joint posterior distribution Pr(***d*** ∣ ***Y***_*qtl*_, ***G***_*qtl*_) while controlling for the SNP distance to the transcription start site (TSS) of the corresponding target gene. As shown in our previous results [14, 22], this approach significantly improves the eQTL discovery.

#### Expected colocalized signals in lipid GWAS

Before conducting the proposed integrative analysis, we first compute the expected fraction of the GWAS hits of blood lipid traits that overlap blood *cis*-eQTLs using the approach described in the Method section. This calculation only requires an approximate estimate of the genome-wide prevalence of causal eQTLs. Here, we show two different approaches for obtaining this estimate.

The first approach utilizes the pre-computed posterior distribution of *cis*-eQTLs and calculates the expected fraction of eQTNs from the posterior distribution by

$$p_{d}=\frac{E(\text{Number}\;\text{of}\;\text{eQTNs})}{p},$$

where the expected number of eQTNs can be conveniently obtained by summing up PIPs for all gene-SNP pairs. For the GTEx whole blood data, we calculate the posterior expected number of eQTNs as 8945.9, and hence, *p*_*d*_ ≈ 1.47 × 10^−3^.

Alternatively, we use a conservative *ad hoc* approach to estimate *p*_*d*_ without a Bayesian analysis of the *cis*-eQTLs. In particular, we note that the GTEx portal reports 6,784 eGenes (i.e., genes harboring *cis*-eQTLs) discovered in the whole blood samples at the 5% FDR level. Assuming that each eGene contains exactly one causal variant, we then estimate *p*_*d*_ ≈ 6,784/6.1 × 10^6^ = 1.11 × 10^−3^. Compared to the previous approach, which is more statistically rigorous, this estimate ignores potential multiple independent eQTNs within an eGene and the uncertainty embedded in the process of eGene discovery (e.g., a non-eGene could be mis-classified and indeed harbor eQTNs). Nevertheless, the two estimates have the same order of magnitude: we observe a causal *cis*-eQTL in approximately 1 out of 1,000 SNPs.

**Fig 4:**
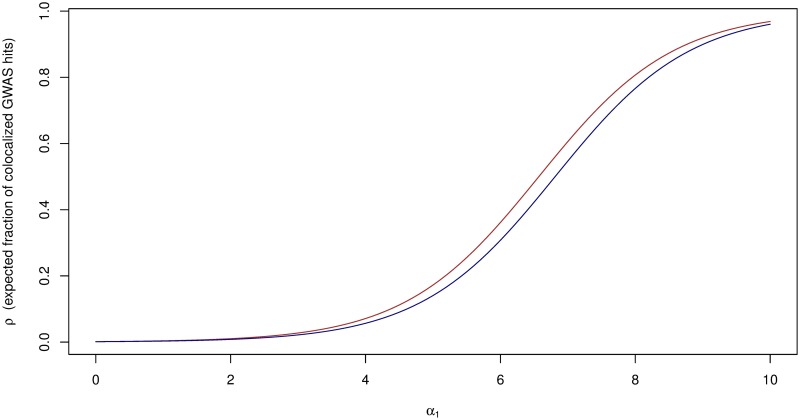
Expected fraction of colocalized GWAS hits in GTEx whole blood *cis*-eQTLs. The red and green curves are computed using the *p*_*d*_ estimates from a model-based and an *ad hoc* approach, respectively. Qualitatively, the two curves are similar. The expected fraction of GWAS hits overlapping *cis*-eQTLs is largely determined by the enrichment parameter *α*_1_: if *α*_1_ → 0, we should expect few colocalization signals, whereas if *α*_1_ is large, a large proportion of GWAS hits are expected to overlap with eQTLs.

We then calculate the expected fraction of GWAS hits overlapping causal eQTLs as a function of enrichment parameter *α*_1_ using the formula (5) for both estimates of *p*_*d*_. The result (shown in Fig 4) indicates that the expected fraction of overlapped signals is largely determined by the level of enrichment. With the current level of eQTL discovery (reflected by *p*_*d*_), we should *not* expect a large fraction of the GWAS hits to overlap with the annotated *cis*-eQTLs unless the enrichment level is reasonably high. For example, even at *α*_1_ ∼ 5, which corresponds to a fold-change at ∼150, the expected fraction of colocalized GWAS signals is still less than 20%—in the case of the genetic variants associated with HDL, the expected number of colocalized signals is ∼10.

#### Enrichment analysis

Next, we apply the proposed multiple imputation procedure to estimate the enrichment level of whole blood *cis*-eQTLs in the GWAS hits of the four lipid traits. As in the analysis of the simulated data sets, we apply the multiple imputation approach for each lipid trait by imputing 25 independent binary eQTL annotations from the joint posterior distribution of the blood *cis*-eQTL data.

**Fig 5:**
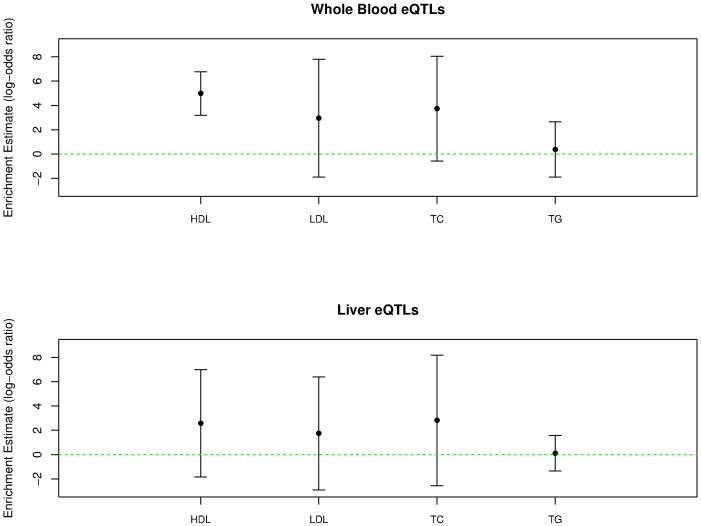
The enrichment estimate of GTEx whole blood and liver *cis*-eQTLs in the GWAS hits of four blood lipid traits. For each trait, the point estimate and the corresponding 95% confidence interval are plotted.

We show the enrichment estimates of blood *cis*-eQTLs in lipid traits and their corresponding 95% confidence intervals in Fig 5. We find that the blood eQTLs are most enriched in the GWAS hits of HDL with point estimate ${\hat{\alpha }}_{1}=4.98$, followed by TC (${\hat{\alpha }}_{1}=3.73$), LDL (${\hat{\alpha }}_{1}=2.95$) and finally TG (${\hat{\alpha }}_{1}=0.38$). The behavior of the proposed enrichment analysis method is very consistent with what we observed in the simulation studies, i.e., imperfect power for enrichment estimates ≤ 4 as we observe that the corresponding 95% confidence intervals cross 0. We further inspect the individual enrichment estimate from each imputed eQTL annotation for each trait (S3 Fig). We find that the enrichment estimates for HDL and TG are quite consistent across all imputed annotation data sets, whereas the estimates for LDL and TC show relatively large variations across imputed annotations. Nevertheless, we observe that all point estimates across all imputed annotations for all 4 traits are positive.

We then plug in the enrichment estimates and calculate the expected fraction of colocalized GWAS hits for each trait from the previously constructed power curves. In summary, we expect that 18%, 3%, 6% and 0.2% of GWAS hits in HDL, LDL, TC and TG overlap with causal *cis*-eQTLs in whole blood. Although the true fractions of overlaps may have large variations due to the uncertainty embedded in the enrichment estimates (as indicated by their large CIs), these estimated expected fractions should reflect the relative difficulty in finding colocalized signals in each lipid trait.

#### Colocalization analysis

Given the enrichment estimates, we proceed to perform the colocalization analysis. Specifically, we apply the Bayesian FDR control procedure [22] implemented in TORUS to identify the LD blocks (defined in Berisa and Pickrell [15]) that harbor at least a single association signal at the 5% FDR level. Ultimately, we identify 72, 64, 78 and 52 genomic loci for HDL, LDL, TC and TG, respectively. Because Teslovich *et al* [7] controlled for the family-wise error rate (FWER) and used a stringent SNP-level genome-wide significance threshold (i.e., 5 × 10^−8^), their reported loci consist of a subset of ours. We further conduct the multi-SNP fine-mapping analysis on each identified GWAS locus, but we find no strong evidence that any of the loci harbors more than one association signal.

Another practical issue arising in the eQTL analysis is that a single SNP can locate in the *cis* regions of multiple genes. Our solution to this problem is to compute an RCP for each LD block with respect to each gene that has at least one *cis* candidate SNP residing in the block. In particular, we construct the SNP prior Eq (7) that is specific to each gene. Consequently, the resulting RCP of the target LD block is also gene specific, which provides a natural way to link the SNP-level GWAS association signals to specific genes. In total, 4,824 genomic region-gene pairs are analyzed across 4 traits.

For comparison, we also perform the colocalization analysis using the existing approaches *eCAVIAR* and *coloc*, and we show the comparisons of the RCPs computed using the different approaches in S4 and S5 Figs. Consistent with what we observe in the simulation studies, *eCAVIAR* yields a highly concordant ranking of potential colocalized signals with *enloc*. However, the numerical values of the RCPs from *eCAVIAR* are generally smaller because of its assumption *α*_1_ = 0. The exception is in the case of TG, where the estimated ${\hat{\alpha }}_{1}\left(=0.38\right)$ is indeed close to 0. The *coloc* analysis is conducted using its default subjective priors for all 4 lipid traits, i.e., *p*_1_ = 10^−4^, *p*_2_ = 10^−4^ and *p*_12_ = 10^−6^. Overall, there is a larger degree of discrepancy in ranking colocalized signals compared to *eCAVIAR* and *enloc*. One of the reasons is that the default priors imply *α*_1_ = 4.6 for all 4 traits, which appear to be inappropriate for LDL, TC and TG. In addition, these priors also indicate a much higher marginal frequency of causal GWAS hits and a much lower marginal frequency of eQTNs compared to our estimations from the data. Although there is generally good concordance of probability quantification for very strong colocalization signals, we find that the severely misspecified priors make the colocalization analysis results less reliable.

In summary, our analysis identifies 21 unique genomic region-gene pairs with an RCP ≥ 0.50. We summarize the results in Table 2. In the context of hypothesis testing, we reject 4 (RCP cutoff of 0.902), 7 (RCP cutoff of 0.832) and 16 (RCP cutoff of 0.639) top-ranked RCP regions at the Bayesian FDR levels of 5%, 10% and 20%, respectively. Within an LD block, we regard an SNP as a contributing colocalized signal if its SCP is ≥ 0.001.

**Table 2:** Identified genomic regions that potentially harbor colocalized association signals of whole blood *cis*-eQTLs and GWAS hits of blood lipid traits. A region is listed if its RCP is ≥ 0.5. We denote the region by ^⋆^ if it is also identified in Teslovich *et al* [7]. The symbol ^†^ indicates that the same gene is linked to the same GWAS hit region in Teslovich *et al* [7].

Trait	Region	Gene	RCP	# of SNPs	Lead SNP	Max SCP
HDL	chr1:109817192-109818530^⋆^	*PSRC1*	0.962	5	rs629301	0.439
HDL	chr2:85537312-85555478	*AC093162.5*	0.845	22	rs10460586	0.340
		*TCF7L1*	0.828	27	rs10460586	0.208
		*ELMOD3*	0.800	22	rs3184780	0.099
HDL	chr3:49971514-50146094	*RBM6*	0.712	22	rs7613875	0.380
HDL	chr6:34548206-34800435^⋆^	*C6orf106*^†^	0.814	35	rs6907508	0.623
HDL	chr9:15303583-15304782^⋆^	*TTC39B*^†^	0.627	2	rs686030	0.580
HDL	chr11:61557803-61623140^⋆^	*TMEM258*	0.832	15	rs102275	0.584
HDL	chr11:122500846-122553139^⋆^	*UBASH3B*^†^	0.639	54	rs60494825	0.036
HDL	chr12:109893156-110042348^⋆^	*MVK*^†^	0.603	45	rs7964021	0.051
HDL	chr19:54796630-54799083^⋆^	*LILRA3*^†^	0.990	3	rs103294	0.979
HDL	chr22:21917450-21980894^⋆^	*UBE2L3*^†^	0.554	39	rs181360	0.052
LDL	chr1:109818306-109818530^⋆^	*PSRC1*	0.901	2	rs629301	0.879
LDL	chr9:136141870-136155000^⋆^	*ABO*^†^	0.582	5	rs550057	0.430
LDL	chr17:8107979-8161149	*C17orf44*	0.708	6	rs8078338	0.637
TC	chr1:109817590-109818530^⋆^	*PSRC1*	0.942	4	rs629301	0.858
TC	chr9:136141870-136155000^⋆^	*ABO*^†^	0.509	4	rs635634	0.327
TC	chr17:8107979-8161149	*C17orf44*	0.745	5	rs8078338	0.671
TC	chr19:49206108-49219459^⋆^	*NTN5*	0.662	7	rs492602	0.177
TC	chr20:34124336-34160840^⋆^	*RPL36P4*	0.745	15	rs2277862	0.494

**Fig 6:**
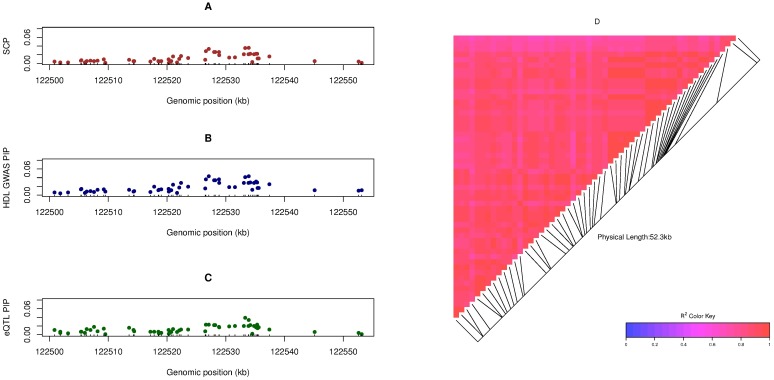
An example of an identified colocalization signal in a high LD region. The region, containing 54 candidate *cis*-eQTL SNPs for gene *UBASH3B*, harbors a GWAS hit for HDL. Panels A, B and C plot the SCPs, eQTL PIPs and GWAS PIPs for each individual SNP, respectively. No single SNP shows a particular high posterior probability in any of the three plots, but the cumulative regional probabilities from all the SNPs are all high. Panel D plots the pairwise LD pattern, measured by *r*^2^, for the 54 SNPs and indicates that all SNPs are tightly linked.

For a small proportion of the identified loci, we find that the colocalized signals can be effectively narrowed down to only a few SNPs. For example, SNP rs103294, a *cis*-eQTL for gene *LILRA3*, has an SCP value of 0.979, showing a strong SNP-level colocalization signal with the GWAS hit of HDL. (Interestingly, our multi-SNP analysis identifies two independent *cis*-eQTLs for *LILRA3*, and the colocalization analysis confidently asserts only one of the eQTLs overlapping with the GWAS hit.) However, the majority of the loci still carry many candidate SNPs due to common LD patterns present in the genetic data of both complex traits and molecular phenotypes. Fig 6 illustrates a colocalized association signal for HDL and the expression of *UBASH3B* in a 52 kb genomic region on chromosome 11. Our analysis identifies 54 SNPs with a joint RCP = 0.645; however, the strongest individual SCP is merely 0.036. Additionally, the SNP-level PIPs for GWAS and *cis*-eQTL associations also exhibit a similar pattern: although there is not a single SNP taking high PIP values, the cumulative PIPs of the region are close to 1 for both GWAS and *cis*-eQTLs. We further compute the pair-wise LD of the 54 member SNPs based on the genotype data from the GTEx samples and confirm that all SNPs are indeed highly correlated.

For the significant loci reported by Teslovich *et al* [7] (labeled by ^⋆^ in Table 2), we compare the genes suggested by our analysis and the reported genes therein. For 8 out of 14 cases, the implicated genes are consistent (labeled by ^†^ in Table 2). Among the other 6 inconsistent cases, 3 involve the genomic region anchored by SNP rs629301, for which our analysis links to *PSRC1* and Teslovich *et al* [7] links to *SORT1* by the more comprehensive molecular evidence presented in Musunuru *et al* [29]. Our examination of the current GTEx analysis results (version 6) reveals that rs629301 shows little to no evidence of association with *SORT1* but very strong evidence of association with *PSRC1* in whole blood; however, in liver, rs629301 shows strong associations with both genes with evidence for *SORT1* being stronger (source: GTEx portal eQTL browser). In addition, the same SNP also shows a strong association with *CELSR2* in liver. We repeat the colocalization analysis using the GTEx liver eQTL data. Not surprisingly, we find that the same genomic region presents the strongest colocalization signals with all 3 genes among all liver-expressed genes, with RCPs = 0.691, 0.684 and 0.675 for *SORT1*, *PSRC1* and *CELSR2*, respectively. The decrease of the RCP values is attributed to the lower eQTL enrichment estimate in liver (${\hat{\alpha }}_{1}=2.567$ with 95% CI [−1.849, 6.984]), which exhibits a considerably larger degree of uncertainty than the whole blood estimate and is likely caused by the insufficient sample size in the current GTEx liver data (sample size of 97 compared to 338 for whole blood). Additionally, we find that the other 3 inconsistent cases can be similarly explained: the blood eQTLs for genes *RPL36P4*, *NTN5* and *TMEM258* all display different association patterns in different types of tissues.

Finally, we note that a single GWAS association can be colocalized to eQTL signals of multiple genes. For example, our analysis indicates that the likely causal HDL variant in the genomic region chr2:85537312-85555478 is possibly associated with the expression levels of 3 different genes (*AC093162.5, TCF7L1* and *ELMOD3*). The case of SNP rs629301 in liver discussed previously is also an example of this type. Although this phenomenon is relatively well known in studies of molecular phenotypes, it certainly makes elucidating the molecular mechanism of causal GWAS variants more challenging.

## Discussion

In this paper, we have proposed a statistically rigorous and computationally efficient analytic framework for performing integrative analyses of GWAS and molecular QTL data and providing quantitative assessments of enrichment and colocalization of their association signals. One of the intrinsic challenges in genetic association analysis is that the resolution of identified association signals is always limited by LD. Consequently, it is generally impossible to pinpoint the causal variants based solely on genetic association analysis, and it imposes a formidable challenge for assessing enrichment and colocalization in the integrative analysis. To address this problem, we formulate a missing data problem and adopt a well-established statistical strategy, i.e., multiple imputation, to fully account for the uncertainty in identifying causal genetic variants for complex traits and molecular phenotypes due to LD. These efforts result in not only more accurate point estimates but also appropriate characterizations of uncertainties of our inference results in the enrichment and colocalization analyses. Particularly, in the colocalization analysis, our theoretical demonstration and the real data example both clearly illustrate that individual SCPs can be unimpressive in high LD regions even if we are confident that the region does harbor a colocalized signal. In light of these findings, we propose and recommend reporting RCPs rather than placing emphasis on colocalization probabilities of individual SNPs.

Compared to the existing methods for colocalization analysis, the most important distinction of our proposed approach is the natural integration of the enrichment estimation. Throughout the paper, we have illustrated the importance of obtaining accurate enrichment estimates on the downstream quantitative evaluations of colocalization. Our main conclusion is that the accurate enrichment estimates based on currently available data may not have an overall large effect on altering the ranking of potential colocalization signals; however, it is critically important for the calibration of the corresponding colocalization probabilities and has a profound impact on the outcome of formal statistical testing procedures. Existing probabilistic model-based approaches typically make explicit assumptions on the enrichment levels of molecular QTLs in the causal GWAS hits (although they may not be presented in the form of enrichment parameters), as we have shown for the cases of *coloc* and *eCAVIAR*. We further hypothesize that all approaches, including empirical methodologies, make implicit assumptions on the enrichment parameters, which can be understood by the hypothetical example of two perfectly linked SNPs discussed in the Method section. For example, if a method determines that the association signals are colocalized in the hypothetical example (without enrichment estimation), it seemingly assumes that the enrichment level is very high (recall that most molecular QTLs are sparse, i.e., *p*_*d*_ ≪ 1, and the RCP → 1 if and only if *α*_1_ → ∞), which is a strong assumption. In summary, we have demonstrated that the enrichment parameter plays a critical role in the colocalization analysis, and we believe that the best strategy to deal with this parameter is to learn it from the observed data, as we have demonstrated throughout.

Importantly, our simulation and real data analyses apparently illustrate the limitations of currently available association data: we have shown that the confidence intervals of enrichment estimates are typically large and the expected fractions of colocalized GWAS signals are only modest, which are consistent with our observations from practice in the field. In particular, we note that most current molecular QTL (e.g., eQTL) studies are conducted with only modest sample sizes due to cost considerations. Although many of these studies successfully identified an abundance of trait-associated genomic loci with large effects, the power required to uncover molecular QTLs with small to modest effects is lacking. Many molecular QTL studies have started scaling up their sample sizes, and novel analytic approaches, e.g., joint eQTL and allelic-specific expression (ASE) analysis [30], have shown great potential in boosting the power of eQTL discovery. Consequently, we expect an elevated estimate of *p*_*d*_ in the near future. Accordingly, based on Eq (5), we anticipate that a higher fraction of GWAS hits overlapping molecular QTLs can be revealed. Similarly, improving the power of GWAS should also help improve discoveries of colocalized signals, which is evident from Eq (6).

Our proposed statistical model and inference procedure are completely general for analyzing two sources of genetic association data. Note that it is statistically equivalent to treating GWAS data as annotations for eQTL mapping. Our choice of presenting eQTL as an annotation is simply motivated by better biological interpretation of the model and our enrichment analysis. It can be shown that when individual-level data are available for both eQTL mapping and GWAS analysis, the choice of annotation should not alter the inference results under the proposed model. More generally, the proposed statistical framework is applicable for analyzing any pair of phenotypes to colocalize the association signals, as in applications demonstrated by Pickrell *et al* [31].

Note that caution should be exercised when attempting to interpret the biological relevance of the identified colocalization signals. In colocalizing an eQTL and a GWAS hit, a seemingly obvious implication is the relevance of the target gene of the eQTL in the disease process. However, as we demonstrated in the analysis of the blood lipid data, there are cases in which other important biological factors should be considered: for example, the relevance of the tissue where the eQTLs are derived from. Although it is generally possible to statistically evaluate the biological relevance of eQTLs from different tissues for a specific complex trait through enrichment analysis, the currently available GTEx data are not satisfactory for this purpose because of the significant variations in sample size across tissues. (We anticipate that this issue will likely be resolved by the end of the GTEx project, and we should re-visit the problem then.) A more elegant solution is to utilize eQTL annotations generated from joint multi-tissue eQTL mapping approaches [32, 33], which enables simultaneous colocalization analysis across multiple tissues. Although conceptually straightforward, the difficulty in implementing a computationally efficient procedure incorporating multi-tissue eQTL data should not be underestimated. We will address this challenge in our future work.

In Testlovich *et al* [7], the authors went to great lengths to establish the biological, clinical and population relevance of genomic loci uncovered in the GWAS, in which integrative genetic association analysis is only a part of the overall process. Despite its own importance, we should acknowledge that integrative analysis of genetic association data is merely a starting point for uncovering the molecular pathway from genetic variants to complex traits.

## Supporting information

S1 Text Supplementary methods and results. (PDF)

S1 Fig Comparison of the simulated summary *Z*-statistics and the observed data from the height GWAS [27]. The overall distributional patterns of *z*-statistics are quite similar. The boxplot indicates that the extreme values from the two distributions are very much comparable; the density plot suggests that the simulated *z*-statistics are more concentrated around 0 and are hence slightly conservative.
(EPS)

S2 FigEnrichment parameter estimates in simulation studies.The proposed multiple imputation approach is compared to three methods utilizing added information that is unattainable in practice and two *ad hoc* imputation methods. The “best case” uses the true association status for both complex traits and molecular QTLs, whereas the “true annotation” utilizes the true association status from molecular QTLs only. The “best snp imputation” annotates the SNP showing the strongest association evidence in an eGene as its sole eQTN. The “mean imputation” annotates each SNP by its PIP. This figure highlights the difficulty in estimating ${\hat{\alpha }}_{1}$ even when additional information is available. It shows the necessity of applying shrinkage to stabilize the point estimates in our simulation setting. It is also evident that the multiple imputation approach outperforms the two *ad hoc* imputation alternatives.(EPS)

S3 Fig Individual estimates and their corresponding 95% confidence intervals from each imputed eQTL annotation data set in the enrichment analysis of the four blood lipid traits. (EPS)

S4 Fig Comparison of colocalization results by *enloc* and *eCAVIAR* in the analysis of blood lipid traits and GTEx whole blood eQTL data. For each trait, we compute the RCPs for each identified locus-gene pair using *eCAVIAR* and *enloc*. The comparison indicates that the two approaches rank candidate loci with high concordance. However, the RCPs computed by *eCAVIAR* are much more conservative because of the assumption *α*_1_ = 0.
(EPS)

S5 Fig Comparison of colocalization results by *enloc* and *coloc* in the analysis of blood lipid traits and GTEx whole blood eQTL data. For each trait, we compute the RCPs for each identified locus-gene pair using *coloc* and *enloc*. Although the two approaches generally agree on the very strong signals, there is considerable discrepancy in both ranking and quantification of the signals.
(EPS)
