# Supplementary material for: Integrating molecular QTL data into genome-wide genetic association analysis: Probabilistic assessment of enrichment and colocalization
Source: PLoS Genet. 2017 Mar 9;13(3):e1006646. doi: 10.1371/journal.pgen.1006646 (PMC5363995; doi:10.1371/journal.pgen.1006646)
Supplement: S1 Text — (PDF) [file pgen.1006646.s001.pdf]

Supplementary Text S1 for "Integrating Molecular QTL Data into Genome-wide Genetic Association Analysis: Probabilistic Assessment of Enrichment and Colocalization"

S.1 Outline of Multiple Imputation Procedure

In this section, we outline the multiple imputation procedure used in the estimation of α_1.

We first analyze the GTEx whole blood *cis*-eQTL using the adaptive DAP algorithm, and obtain the joint distribution of $\Pr(\boldsymbol{d} \mid \boldsymbol{Y}_{qtl}, \boldsymbol{G}_{qtl})$ for each gene. Subsequently, we create $m = 25$ imputed annotations by independent sampling from the corresponding posterior distribution of each gene. We then perform the enrichment estimate for each imputed data set using the EM-DAP1 algorithm implemented in the software package TORUS. In the end, we obtain a point estimate of α_1, namely $\hat{\alpha}_1^{(i)}$, and its standard error, $\sigma^{(i)}$ for each imputed data set i.

The multiple imputation procedure combines the estimates from individual imputed data sets in the following way. First, the overall point estimate $\hat{\alpha}_1$ is simply given by

$$\hat{\alpha}_1 = \frac{1}{m} \sum_{i=1}^{m} \hat{\alpha}_1^{(i)}. \tag{1}$$

The variance of the point estimate is then computed by

$$\sigma_{MI}^2 = \sigma_B^2 + (1 + 1/m)\sigma_W^2, \tag{2}$$

where

$$\sigma_B^2 = \frac{1}{m} \sum_{i=1}^{m} (\sigma^{(i)})^2, \tag{3}$$

and

$$\sigma_W^2 = \frac{1}{m-1} \sum_{i=1}^{m} (\hat{\alpha}_1^{(i)} - \hat{\alpha}_1)^2. \tag{4}$$

A more detailed reference on multiple imputation procedure can be found in Schafer [1].

S.2 Adaptive Shrinkage in Enrichment Estimation

As discussed in the main text, the lack of strongly colocalized signals leads to unstable enrichment estimate. Here we propose a data-driven empirical approach to remedy this issue. The general statistical idea is to trade off the variance of the enrichment estimate against bias to achieve an overall accurate prior estimate for the downstream fine-mapping and colocalization analyses.

In the EM algorithm that we previously detailed [2, 3], we showed that the M-step is equivalent to fitting a logistic regression model, which, in our context, regress the expected association status estimated in the E-step on the imputed eQTL annotation for each SNP. To apply the shrinkage on the estimate, we apply an l_2 penalty with a constant shrinkage parameter λ in fitting the logistic regression for each M-step, which is equivalent to assuming a normal prior $\mathrm{N}(0, 1/\lambda)$ on α_1.

We determine the shrinkage parameter in a data-adaptive way. Intuitively, a larger shrinkage is desired if the data indicates more severe sparsity of overlapping association signals. If both $\boldsymbol{\gamma}$ and $\boldsymbol{d}$ are observed, we can construct a 2×2 contingency table and α_1 and its variance can be estimated in a standard way. In particular, the variance of the log-odds ratio estimate can be used as a measure for our purpose: e.g., the variance is small if all 4 cells have sufficient counts, whereas if the count of one cell is small, the overall variance of the log-odds ratio estimate is large. For each imputed data set, $\boldsymbol{d}$ is indeed observed but $\boldsymbol{\gamma}$ remains latent. We therefore estimate the cell count of the hypothetical 2×2 contingency table by computing the PIP of each SNP in GWAS using the DAP-1 algorithm and assuming $\alpha_1 = 0$. More specifically, let η_i denote the indicator if the SNP i is a molecular QTN in the imputed data set and let p_i denote the resulting PIP from the DAP-1 algorithm. Assuming there are total p candidate SNPs, we estimate the 4 cell counts by

$$\begin{aligned}
E_{00} &= \sum_{i=1}^{p} (1 - p_i)(1 - \eta_i), \\
E_{01} &= \sum_{i=1}^{p} (1 - p_i)\eta_i, \\
E_{10} &= \sum_{i=1}^{p} p_i(1 - \eta_i), \\
E_{11} &= \sum_{i=1}^{p} p_i \eta_i.
\end{aligned} \tag{5}$$

Finally, we set the shrinkage parameter λ by

$$\lambda = \left(\frac{1}{E_{00}} + \frac{1}{E_{01}} + \frac{1}{E_{10}} + \frac{1}{E_{11}} \right), \tag{6}$$

which is the variance of the log-odds ratio estimate if true cell counts are indeed observed (instead of estimated). This shrinkage parameter has a natural interpretation and quantitatively reflects the imbalance of the unobserved 2×2 contingency table.

S.3 Derivation of SNP-level Colocalization Probability

The SNP-level colocalization probability can be computed by noting the relationship between the posterior probabilities $\Pr(\gamma_i = 1, \delta_i = 1 \mid \boldsymbol{y}, \boldsymbol{G}, \boldsymbol{Y}_{qtl}, \boldsymbol{G}_{qtl}, \hat{\boldsymbol{\alpha}})$ and $\Pr(\gamma_i = 1, \delta_i = 1 \mid \boldsymbol{y}, \boldsymbol{G}, \boldsymbol{Y}_{qtl}, \boldsymbol{G}_{qtl}, \hat{\boldsymbol{\alpha}})$ and their relationship to the marginal PIP in the GWAS data, i.e.,

$$\begin{aligned} &\Pr(\gamma_i = 1 \mid \boldsymbol{y}, \boldsymbol{G}, \boldsymbol{Y}_{qtl}, \boldsymbol{G}_{qtl}, \hat{\boldsymbol{\alpha}}) = \\ &\qquad \Pr(\gamma_i = 1, \delta_i = 1 \mid \boldsymbol{y}, \boldsymbol{G}, \boldsymbol{Y}_{qtl}, \boldsymbol{G}_{qtl}, \hat{\boldsymbol{\alpha}}) + \Pr(\gamma_i = 1, \delta_i = 0 \mid \boldsymbol{y}, \boldsymbol{G}, \boldsymbol{Y}_{qtl}, \boldsymbol{G}_{qtl}, \hat{\boldsymbol{\alpha}}), \\ &\text{and} \\ &\frac{\Pr(\gamma_i = 1, \delta_i = 1 \mid \boldsymbol{y}, \boldsymbol{G}, \boldsymbol{Y}_{qtl}, \boldsymbol{G}_{qtl}, \hat{\boldsymbol{\alpha}})}{\Pr(\gamma_i = 1, \delta_i = 0 \mid \boldsymbol{y}, \boldsymbol{G}, \boldsymbol{Y}_{qtl}, \boldsymbol{G}_{qtl}, \hat{\boldsymbol{\alpha}})} = \frac{e^{\hat{\alpha}_1} + e^{\hat{\alpha}_0 + \hat{\alpha}_1}}{1 + e^{\hat{\alpha}_0 + \hat{\alpha}_1}} \cdot \frac{\delta_i}{1 - \delta_i}. \end{aligned} \tag{7}$$

Solving this linear system yields the following expression for SNP-level colocalization probability,

$$\begin{aligned} &\Pr(\gamma_i = 1, \delta_i = 1 \mid \boldsymbol{y}, \boldsymbol{G}, \boldsymbol{Y}_{qtl}, \boldsymbol{G}_{qtl}, \hat{\boldsymbol{\alpha}}) \\ &= \Pr(\gamma_i = 1 \mid \boldsymbol{y}, \boldsymbol{G}, \boldsymbol{Y}_{qtl}, \boldsymbol{G}_{qtl}, \hat{\boldsymbol{\alpha}}) \Bigg/ \left[1 + \frac{1 - \delta_i}{\delta_i} \cdot \frac{1 + e^{\hat{\alpha}_0 + \hat{\alpha}_1}}{e^{\hat{\alpha}_1} + e^{\hat{\alpha}_0 + \hat{\alpha}_1}} \right]. \end{aligned} \tag{8}$$

S.4 Connections to coloc Model

Here we compare the statistical approach *coloc* to our proposed method. Specifically, we show that *coloc* can be viewed as a rough approximation and a special case to our general integrative analysis approach.

The method *coloc* requires pre-defining three SNP-level prior probabilities p_1, p_2 and p_{12}. Using the

notations of this paper, these three quantities can be formulated as

$$p_1 := \Pr(\gamma_i = 1, d_i = 0) = \frac{\exp(\alpha_0)}{1 + \exp(\alpha_0)} \cdot [1 - \Pr(d_i = 1)]\,,$$
$$p_2 := \Pr(\gamma_i = 0, d_i = 1) = \frac{1}{1 + \exp(\alpha_0 + \alpha_1)} \cdot \Pr(d_i = 1),$$
$$p_{12} := \Pr(\gamma_i = 1, d_i = 1) = \frac{\exp(\alpha_0 + \alpha_1)}{1 + \exp(\alpha_0 + \alpha_1)} \cdot \Pr(d_i = 1).$$

Additionally, the prior probability $\Pr(\gamma_i = 0, d_i = 0) = 1 - p_1 - p_2 - p_{12}$ can be trivially computed and represented by p_0. In comparison, we explicitly estimate α_0 and α_1 from the GWAS data. Although we do not directly utilize the prior probability of a QTN, $\Pr(d_i = 1)$ (rather, the inferred posterior probability $\Pr(d_i = 1 \mid \boldsymbol{Y}_{qtl}, \boldsymbol{G}_{qtl})$ is applied throughout our inference procedure), this very quantity can be straightforwardly estimated from the eQTL data, $(\boldsymbol{Y}_{qtl}, \boldsymbol{G}_{qtl})$, using the EM-DAP1 algorithm [4].

Similar to our RCP quantification, the *coloc* method considers the existence of a colocalized GWAS and molecular QTL signal within a LD block. Importantly the *coloc* model makes an explicit assumption that there is at most a single GWAS hit and/or a single QTN within the locus of interest, which enables highly efficient approximate computation for the RCP. Here we show that, given the simplifying assumption and the pre-specified priors for p_1, p_2 and p_{12}, *coloc* yields identical result of RCP as the proposed method given p_1, p_2 and p_{12}.

Suppose that there are m SNPs in the LD block of interest and let the binary m-vectors $\boldsymbol{\gamma}_l$ and $\boldsymbol{d}_l$ denote their association status with respect to the complex trait and the molecular phenotype, respectively. Because GWAS and Molecular QTL data are obtained from non-overlapping samples, it follows that

$$\begin{aligned} \Pr(\boldsymbol{\gamma}_l, \boldsymbol{d}_l \mid \boldsymbol{y}, \boldsymbol{G}, \boldsymbol{Y}_{qtl}, \boldsymbol{G}_{qtl}) &\propto \Pr(\boldsymbol{\gamma}_l, \boldsymbol{d}_l)\, P(\boldsymbol{y} \mid \boldsymbol{G}, \boldsymbol{\gamma}_l)\, P(\boldsymbol{Y}_{qtl} \mid \boldsymbol{G}_{qtl}, \boldsymbol{d}) \\ &\propto \left(\prod_i \Pr(\gamma_i, d_i)\right) P(\boldsymbol{y} \mid \boldsymbol{G}, \boldsymbol{\gamma}_l)\, P(\boldsymbol{Y}_{qtl} \mid \boldsymbol{G}_{qtl}, \boldsymbol{d}) \end{aligned} \tag{9}$$

Assuming that SNP l_i is *the* colocalized association signal, it follows from our proposed model that

$$\begin{aligned}
&\frac{\Pr(\gamma_{l_i}=1, d_{l_i}=1, \boldsymbol{\gamma}_{l\setminus i}=\mathbf{0}, \boldsymbol{d}_{l\setminus i}=\mathbf{0} \mid \boldsymbol{y}, \boldsymbol{G}, \boldsymbol{Y}_{qtl}, \boldsymbol{G}_{qtl})}{\Pr(\boldsymbol{\gamma}_l=\mathbf{0}, \boldsymbol{d}_l=\mathbf{0} \mid \boldsymbol{y}, \boldsymbol{G}, \boldsymbol{Y}_{qtl}, \boldsymbol{G}_{qtl})} \\
&= \frac{\Pr(\gamma_{l_i}=1, d_{l_i}=1)\Pr(\boldsymbol{\gamma}_{l\setminus i}=0, \boldsymbol{d}_{l\setminus i}=0)}{\Pr(\gamma_{l_i}=0, d_{l_i}=0)\Pr(\boldsymbol{\gamma}_{l\setminus i}=0, \boldsymbol{d}_{l\setminus i}=0)} \cdot \frac{P(\boldsymbol{y} \mid \boldsymbol{G}, \gamma_i=1, \boldsymbol{\gamma}_{l\setminus i}=\mathbf{0})}{P(\boldsymbol{y} \mid \boldsymbol{G}, \boldsymbol{\gamma}_l=\mathbf{0})} \cdot \frac{P(\boldsymbol{Y}_{qtl} \mid \boldsymbol{G}_{qtl}, d_{l_i}=1, \boldsymbol{d}_{l\setminus i}=\mathbf{0})}{P(\boldsymbol{Y}_{qtl} \mid \boldsymbol{G}_{qtl}, \boldsymbol{d}_l=\mathbf{0})} \\
&= \frac{\Pr(\gamma_{l_i}=1 \mid d_{l_i}=1)\Pr(d_{l_i}=1)}{\Pr(\gamma_{l_i}=0 \mid d_{l_i}=0)\Pr(d_{l_i}=0)} \cdot \frac{P(\boldsymbol{y} \mid \boldsymbol{G}, \gamma_i=1, \boldsymbol{\gamma}_{l\setminus i}=\mathbf{0})}{P(\boldsymbol{y} \mid \boldsymbol{G}, \boldsymbol{\gamma}_l=\mathbf{0})} \cdot \frac{P(\boldsymbol{Y}_{qtl} \mid \boldsymbol{G}_{qtl}, d_{l_i}=1, \boldsymbol{d}_{l\setminus i}=\mathbf{0})}{P(\boldsymbol{Y}_{qtl} \mid \boldsymbol{G}_{qtl}, \boldsymbol{d}_l=\mathbf{0})} \\
&\approx \frac{p_{12}}{p_0} \cdot \mathrm{BF}_{l_i,\mathrm{gwas}} \cdot \mathrm{BF}_{l_i,\mathrm{qtl}},
\end{aligned} \tag{10}$$

where the marginal likelihood ratios are approximated by the Bayes factors of single-SNP association models for the complex trait and molecular phenotype, respectively.

Under the constraint imposed by the simplifying assumption, all possible configurations of $(\boldsymbol{\gamma}_l, \boldsymbol{d}_l)$ can be enumerated, of which the corresponding posterior probability can be similarly computed as (10). For example,

$$\begin{aligned}
&\frac{\Pr(\gamma_{l_i}=1, \boldsymbol{\gamma}_{l\setminus i}=\mathbf{0}, \boldsymbol{d}_l=\mathbf{0} \mid \boldsymbol{y}, \boldsymbol{G}, \boldsymbol{Y}_{qtl}, \boldsymbol{G}_{qtl})}{\Pr(\boldsymbol{\gamma}_l=\mathbf{0}, \boldsymbol{d}_l=\mathbf{0} \mid \boldsymbol{y}, \boldsymbol{G}, \boldsymbol{Y}_{qtl}, \boldsymbol{G}_{qtl})} \\
&\approx \frac{p_1}{p_0} \cdot \mathrm{BF}_{l_i,\mathrm{gwas}},
\end{aligned} \tag{11}$$

$$\begin{aligned}
&\frac{\Pr(\boldsymbol{\gamma}_l=\mathbf{0}, d_{l_i}=1, \boldsymbol{d}_{l\setminus i}=\mathbf{0} \mid \boldsymbol{y}, \boldsymbol{G}, \boldsymbol{Y}_{qtl}, \boldsymbol{G}_{qtl})}{\Pr(\boldsymbol{\gamma}_l=\mathbf{0}, \boldsymbol{d}_l=\mathbf{0} \mid \boldsymbol{y}, \boldsymbol{G}, \boldsymbol{Y}_{qtl}, \boldsymbol{G}_{qtl})} \\
&\approx \frac{p_2}{p_0} \cdot \mathrm{BF}_{l_i,\mathrm{qtl}},
\end{aligned} \tag{12}$$

and,

$$\begin{aligned}
&\frac{\Pr(\gamma_{l_i}=1, d_{l_j}=1, \boldsymbol{\gamma}_{l\setminus i}=\mathbf{0}, \boldsymbol{d}_{l\setminus j}=\mathbf{0}, i \neq j \mid \boldsymbol{y}, \boldsymbol{G}, \boldsymbol{Y}_{qtl}, \boldsymbol{G}_{qtl})}{\Pr(\boldsymbol{\gamma}_l=\mathbf{0}, \boldsymbol{d}_l=\mathbf{0} \mid \boldsymbol{y}, \boldsymbol{G}, \boldsymbol{Y}_{qtl}, \boldsymbol{G}_{qtl})} \\
&\approx \frac{p_1}{p_0} \cdot \frac{p_2}{p_0} \cdot \mathrm{BF}_{l_i,\mathrm{gwas}} \cdot \mathrm{BF}_{l_j,\mathrm{qtl}},
\end{aligned} \tag{13}$$

In the end, *coloc* computes the RCP for the locus of interest by

$$\mathrm{RCP}_{\mathrm{coloc}} = \frac{1}{C} \cdot \frac{p_{12}}{p_0} \sum_{i=1}^{m} \left(\mathrm{BF}_{l_i,\mathrm{gwas}} \cdot \mathrm{BF}_{l_i,\mathrm{qtl}}\right), \tag{14}$$

where C denotes the normalizing constant and is computed by

$$C = \sum_{(\boldsymbol{\gamma}_l, \boldsymbol{d}_l) \in \Omega} \frac{\Pr(\boldsymbol{\gamma}_l, \boldsymbol{d}_l \mid \boldsymbol{Y}, \boldsymbol{G}, \boldsymbol{Y}_{qtl}, \boldsymbol{G}_{qtl})}{\Pr(\boldsymbol{\gamma}_l=\mathbf{0}, \boldsymbol{d}_l=\mathbf{0} \mid \boldsymbol{Y}, \boldsymbol{G}, \boldsymbol{Y}_{qtl}, \boldsymbol{G}_{qtl})},$$

and the set Ω denotes the eligible $(\boldsymbol{\gamma}_l, \boldsymbol{d}_l)$ configurations under the constraint. Additionally, *coloc* approximates $p_0 \approx 1$, and expression (14) becomes identical to what is used in [5].

In summary, we have shown that the *coloc* approach can be derived as a special case from our proposed general framework with the following added assumptions,

1. the prior values are pre-defined through p_1, p_2 and p_{12}
2. there is at most one causal GWAS variant in the region of interest
3. there is at most one causal molecular QTN in the region of interest

Next, we use simulated data to examine the effect of prior misspecification on the colocalization analysis. The default priors provided by *coloc* are $p_1 = 10^{-4}, p_2 = 10^{-4}$ and $p_{12} = 10^{-6}$, which correspond to $\alpha_0 = -9.2$ and $\alpha_1 = 4.6$ in our parameterization. These default prior roughly match the simulated GWAS data, however the frequency of eQTNs suggested by the priors is much lower than the true value used in the simulation, as well as our model estimates. Primarily due to the conservative eQTL prior, the *coloc* annotates a much smaller set of eQTLs in our simulated data. We find that this results in an inferior performance in ranking potential colocalized signals as indicated by the ROC curve (Fig. 1). In particular, at a given false positive rate threshold, we find much fewer colocalized signals are identified comparing to the proposed approach. In the application of hypothesis testing, we find that the type I errors are inflated, in some cases severely, indicating that the posterior colocalization probabilities are poorly calibrated. For example, at 5%, 10% FDR levels, the realized FDRs are 14% and 20%, respectively.

S.5 Multi-SNP Analysis with Adaptive DAP Algorithm using Summary-level Statistics

For practical and/or privacy considerations, many GWAS data are made available with only summary-level statistics, typically in the forms of single-SNP testing p-values or z-scores. In this section, we discuss the analytic strategy to perform proposed analysis using only summary-level statistics from GWAS. Many authors have demonstrated that the SNP-level PIP in GWAS, $\Pr(\gamma_i = 1 \mid \boldsymbol{y}, \boldsymbol{G}, \boldsymbol{Y}_{qtl}, \boldsymbol{G}_{qtl}, \hat{\boldsymbol{\alpha}})$, can be *approximated* from the summary-level statistics obtained from single-SNP association testing results [6,7]. Here we show their results extend to the application of the adaptive DAP algorithm.

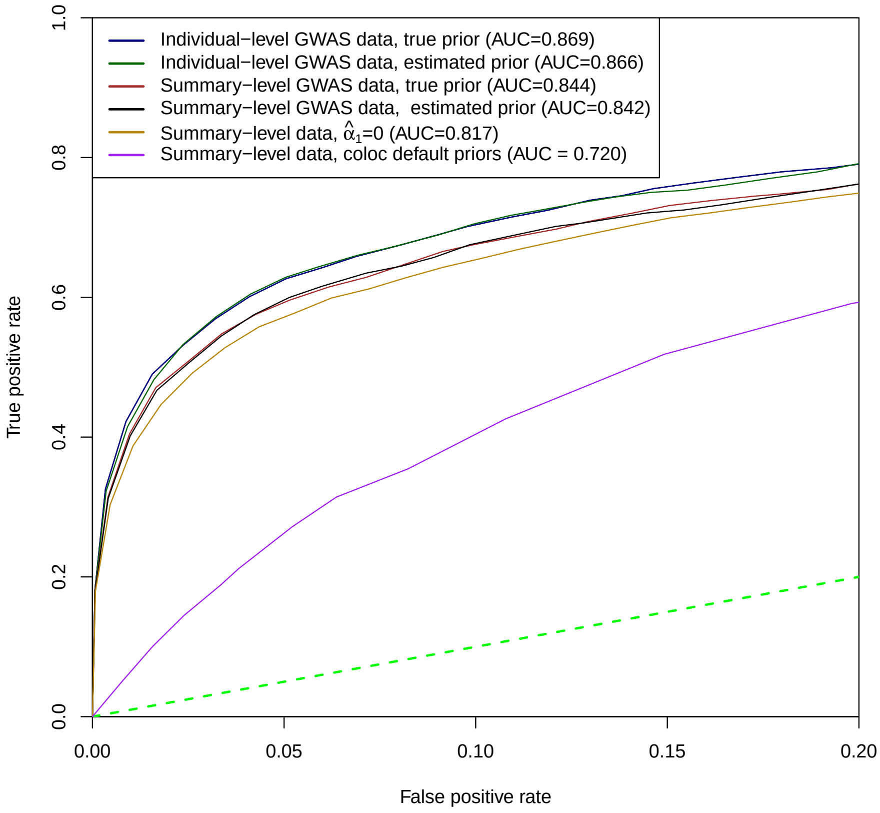

Fig. 1. ROC curve generated from *coloc* using its default priors in comparison to other approaches applied in the simulation study.

In the case where individual-level genotype data are unavailable, the main computational difficulty in applying adaptive DAP algorithm lies in calculating the marginal likelihood, i.e., Bayes factor, for any given value of $\boldsymbol{\gamma}$. There are primarily two types of approaches to circumvent this difficulty. This first type attempts to derive the likelihood by treating summary-level statistics as the sole observed data: the results by Chen *et al* [8] (Equation (3)) and Zhu and Stephens [7] (Equation (2.10)) provide suitable approximations that are accurate and easy to compute. The second type of approach is based on the trivial fact that given the summary-level statistics, namely z-scores from the single-SNP analysis, and the exact matrix $\boldsymbol{G}'\boldsymbol{G}$, the exact likelihood function can be computed for any given $\boldsymbol{\gamma}$ as the individual level data, $(\boldsymbol{y}, \boldsymbol{G})$, are provided. The $p \times p$ matrix, $\boldsymbol{G}'\boldsymbol{G}$, characterizes the correlations between genotypes among candidate SNPs. When the full matrix from the samples is not available, we can estimate it based

on the LD patterns observed in an appropriate population genotype panel.

Our current implementation in the adaptive DAP algorithm takes the second approach and requires single-SNP testing z-values and either an estimate or exact matrix of $\boldsymbol{G}'\boldsymbol{G}$. (We also plan to implement the approximation by [8].)

S.6 Caveat of Mean Imputation for Enrichment Analysis

An often-applied imputation strategy is to impute the missing values by their corresponding expectations, which is commonly named as "mean imputation". Within our proposed modeling framework, a mean imputation strategy would simply treat the PIPs from the molecular QTL analysis as the observed annotations. The general drawbacks of the mean imputation have been thoroughly discussed in the statistical literature. Here we focus on a particular observation from the simulation studies: the mean imputation consistently overestimates the α_1 values and sometimes the degree of the overestimation is high. Among many contributing factors to this phenomenon, we illustrate one factor, for which we name as the "scaling factor", to explain the observed overestimation.

If $\boldsymbol{d}$ is indeed observed, fitting the logistic regression model

$$\log \left[\frac{\Pr(\gamma_i = 1)}{\Pr(\gamma_i = 0)} \right] = \alpha_0 + \alpha_1 d_i. \tag{15}$$

leads to an unbiased estimator of α_1. The mean imputation replaces each d_i value by its expectation δ_i from the QTL analysis. Because δ_i's are valid probabilities, they are always shrunk towards 0.5, regardless of the true value of d_i. This consequently narrows the scale of the covariate in the logistic model, which requires the regression coefficient α_1 to be "scaled-up", i.e., overestimated, accordingly. Consider an extreme example where all 0's and 1's are replaced by 0.1's and 0.9's, respectively, and α_1 is positively estimated when $\boldsymbol{d}$ is observed. With the same input $\boldsymbol{\gamma}$ values, the ratio of the α_1 estimates in the re-scaled vs. original regression model is exactly 1.25, and the difference between the two becomes more noticeable if the true α_1 is relatively large.

The scaling factor, at least in part, explains the inflation of the α_1 estimate. It also should be noted that the degree of the overestimation is related the overall accuracy of the mean imputation: if the imputation is extremely accurate, i.e., δ_i's become very close to their true binary values, the effect of the scaling factor can be negligible.

References

1. Schafer JL (1997) Analysis of incomplete multivariate data. CRC press.

2. Wen X, Luca F, Pique-Regi R (2015) Cross-population joint analysis of eqtls: Fine mapping and functional annotation. PLOS Genetics 11: e1005176.

3. Wen X (2016) Molecular qtl discovert incorporating genomic annotations using bayesian false discovery rate control. Annals of Applied Statistics .

4. Wen X, Lee Y, Luca F, Pique-Regi R (2016) Efficient integrative multi-snp association analysis via deterministic approximation of posteriors. The American Journal of Human Genetics 98: 1114–1129.

5. Giambartolomei C, Vukcevic D, Schadt EE, Franke L, Hingorani AD, et al. (2014) Bayesian test for colocalisation between pairs of genetic association studies using summary statistics. PLoS Genet 10: e1004383.

6. Kichaev G, Yang WY, Lindstrom S, Hormozdiari F, Eskin E, et al. (2014) Integrating functional data to prioritize causal variants in statistical fine-mapping studies. PLOS Genetics 10: e1004722.

7. Zhu X, Stephens M (2016) Bayesian large-scale multiple regression with summary statistics from genome-wide association studies. bioRxiv : 042457.

8. Chen W, Larrabee BR, Ovsyannikova IG, Kennedy RB, Haralambieva IH, et al. (2015) Fine mapping causal variants with an approximate bayesian method using marginal test statistics. Genetics 200: 719–736.
